# Gene regulatory networks for compatible versus incompatible grafts identify a role for SlWOX4 during junction formation

**DOI:** 10.1093/plcell/koab246

**Published:** 2021-10-05

**Authors:** Hannah Thomas, Lisa Van den Broeck, Ryan Spurney, Rosangela Sozzani, Margaret Frank

**Affiliations:** 1 School of Integrative Plant Science, Cornell University, Ithaca, New York 14850, USA; 2 Department of Plant and Microbial Biology, North Carolina State University, Raleigh, North Carolina 27695, USA; 3 Department of Electrical and Computer Engineering, North Carolina State University, Raleigh, North Carolina 27695, USA

## Abstract

Grafting has been adopted for a wide range of crops to enhance productivity and resilience; for example, grafting of Solanaceous crops couples disease-resistant rootstocks with scions that produce high-quality fruit. However, incompatibility severely limits the application of grafting and graft incompatibility remains poorly understood. In grafts, immediate incompatibility results in rapid death, but delayed incompatibility can take months or even years to manifest, creating a significant economic burden for perennial crop production. To gain insight into the genetic mechanisms underlying this phenomenon, we developed a model system using heterografting of tomato (*Solanum lycopersicum*) and pepper (*Capsicum annuum*). These grafted plants express signs of anatomical junction failure within the first week of grafting. By generating a detailed timeline for junction formation, we were able to pinpoint the cellular basis for this delayed incompatibility. Furthermore, we inferred gene regulatory networks for compatible self-grafts and incompatible heterografts based on these key anatomical events, which predict core regulators for grafting. Finally, we examined the role of vascular development in graft formation and uncovered SlWOX4 as a potential regulator of graft compatibility. Following this predicted regulator up with functional analysis, we show that *Slwox4* homografts fail to form xylem bridges across the junction, demonstrating that indeed, *SlWOX4* is essential for vascular reconnection during grafting, and may function as an early indicator of graft failure.

## Teaser

Gene regulatory networks for compatible self-grafted and incompatible heterografted pepper/tomato plants identify a role for SlWOX4 during junction formation.

## Introduction

Plants have robust systems for self-regeneration following wounding (Savatin et al., 2014; Ikeuchi et al., 2019). Grafting is an ancient agricultural approach that relies on the innate capacity of plants to undergo self-repair. Grafting surgically joins independent root and shoot systems, creating a dual plant system that expresses superior traits on either half of the junction. This approach has been strategically adopted in a wide range of species to boost crop productivity and resilience (Mudge et al., 2009; Gaut et al., 2019). Successful grafts are dependent on the formation of the graft junction, a dynamic anatomical connector that unites the rootstock and scion.

While survival has recently been equated with graft compatibility, the classic definition for compatible combinations states that both nonvascular (cortex/pith, epidermis) and vascular connections must be made between the scion and stock (Proebsting, 1928). Within the Solanaceae, potato (*Solanum tuberosum*), tobacco (*Nicotiana tabacum*), and eggplant (*Solanum melongena*) are routinely grafted with tomato (*Solanum lycopersicum*) for horticultural purposes (Lee and Oda, 2010; Dawson, 1942). Unlike other Solanaceous plants, Capsicum species (peppers) are only graft compatible with other Capsicum species (Kawaguchi et al., 2008; Lee and Oda, 2010), and tomato and pepper (*Capsicum annuum*) graft combinations have been described as “severely” incompatible (Kawaguchi et al., 2008). The capacity for an incompatible graft to survive for months, or even years in perennial crops, without forming a successful vascular connection is referred to as delayed incompatibility (Argles, 1937). Stunted root and shoot growth, the formation of suckers or adventitious roots, and large, bulging graft junctions are all symptoms of delayed incompatibility (Eames and Cox, 1945; Copes, 1980; Zarrouk et al., 2006). Graft combinations with delayed incompatibility eventually succumb to their mechanical weakness and break at the graft junction, presenting severe challenges for commercial growers (Kawaguchi et al., 2008).

Despite the long history and wide-spread use of grafting, only eight genes have been directly implicated in junction formation. These genes are involved in cell proliferation and vascular specification (Asahina et al., 2011; Pitaksaringkarn et al., 2014; Matsuoka et al., 2018, 2021; Melnyk et al., 2018; Notaguchi et al., 2020).

Given the essential role of vascular reconnection during graft formation, genes involved in the relatively well-characterized process of cambium-xylem maintenance serve as promising developmental regulators of junction formation. Vascular development in *Arabidopsis thaliana* roots is regulated by a dynamic transcription factor (TF) network coordinated with hormonal inputs. CLAVATA3/EMBRYO SURROUNDING REGION-RELATED (CLE) peptides 41 and 44 encode identical peptides that act as TRACHEARY ELEMENT DIFFERENTIATION INHIBITORY FACTORs (TDIFs), which are produced in the phloem and bind to the PHLOEM INTERCALATED WITH XYLEM (PXY) cambial receptor (Ito, 2006; Smit et al., 2020). Activated PXY is involved in the maintenance of cambial cells by promoting WUSCHEL-RELATED HOMEOBOX 4 (WOX4) and WOX14 (Fisher and Turner, 2007; Etchells and Turner, 2010; Hirakawa et al., 2010; Suer et al., 2011; Etchells et al., 2013; Han et al., 2018). Downstream of WOX14, there are important cambial regulators such as KNOTTED-LIKE FROM ARABIDOPSIS THALIANA (KNAT1) and LOB DOMAIN-CONTAINING PROTEIN 4 (LBD4; Mele et al., 2003). PXY also represses xylem differentiation factors such as VASCULAR-RELATED NAC-DOMAIN 6 (VND6), VND7, and NAC SECONDARY WALL THICKENING PROMOTING FACTORs (NSTs) via brassinosteroid signaling (Kubo et al., 2005; Zhong et al., 2007; Mitsuda et al., 2007; Kondo et al., 2015; Turco et al., 2019).

In line with the hypothesis that genes involved in xylem-cambial maintenance play a role during junction formation, several core regulators for vascular genesis were identified in recent graft transcriptome studies (Melnyk et al., 2018; Xie et al., 2019). Moreover, these studies uncovered a subset of genes that were asymmetrically expressed either in the scion or the rootstock during graft formation, which lead to an as-yet untested hypothesis that asymmetric expression across the graft interface drives junction formation (Melnyk et al., 2018; Xie et al., 2019).

In this study, we investigate the molecular mechanisms underlying compatible versus incompatible grafts by connecting anatomical processes with predicted regulatory interactions. Through anatomical, biophysical, and genetic characterization, we have established tomato and pepper as a model system for studying graft incompatibility. To our knowledge, only one study has employed regulatory networks to identify genes involved in graft formation (Xie et al., 2019). Next, we utilized Bayesian inference and regression analyses to expand our understanding of species-specific genetic responses, which regulate the conserved process of junction formation (Prill et al., 2010; de Luis Balaguer and Sozzani, 2017; de Luis Balaguer et al., 2017; Clark et al., 2019; Smet et al., 2019). We then identified orthologs of known genetic factors involved in vascular development, which uncovered SlWOX4 as a potential regulator of graft compatibility. In line with this hypothesis, we show that *Slwox4* homografts fail to form xylem bridges across the junction. These functional analyses demonstrate that indeed, *SlWOX4* is essential for vascular reconnection during grafting, and may function as an early indicator of graft failure.

## Results

### Tomato and pepper exhibit delayed incompatibility

**Figure 1:**
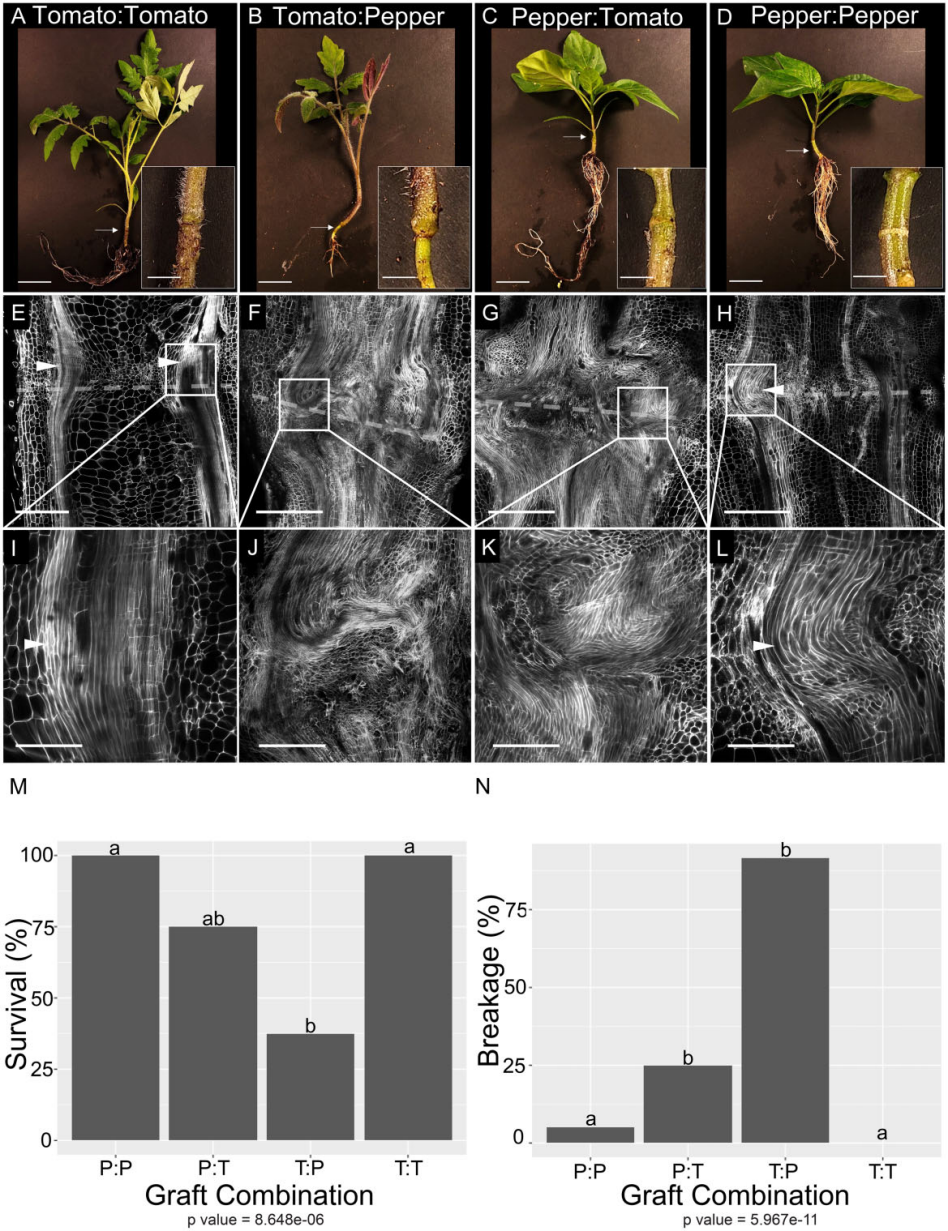
Heterografted tomato and pepper plants show severe vascular patterning defects, reduced viability, and biomechanical failure 30 DAG. A–D, Representative images of self-grafted tomato (A), heterografted tomato:pepper (B), pepper:tomato (C), and self-grafted pepper (D) plants taken 30 DAG. White arrows indicate graft junctions. E–L, High-resolution confocal imaging of vascular anatomy for self-grafted tomato (E and I), heterografted tomato:pepper (F and J), and pepper:tomato (G and K), and self-grafted pepper (H and L) plants taken at 30 DAG. Tissues were stained with PI to visualize cell walls, and cleared in methyl salicylate. White arrowheads point to xylem bridges. Dashed lines represent the graft site. (M and N) Heterografts exhibited significantly reduced viability relative to self-grafted plants (M), and higher breakage along the graft site during bend tests (N). *P*-values under graphs shown from Fisher’s exact test. Different letters indicate significant differences in the graft combinations (pairwise comparisons using Fisher’s exact test, *P *<* *0.05, *P*-values shown in Supplemental Data Set 1). P:P = pepper:pepper graft, T:T = tomato:tomato graft, P:T = pepper:tomato graft, T:P = tomato:pepper graft, PI = Propidium Iodide. N = 3 (A–L), *n* = 12–18 (M, N). Scale bars = 2 cm (A–D), 1 cm (E–H), 400 µm (I–L).

To investigate the developmental regulation of graft compatibility, we developed a genetically tractable heterografting system between *Solanum lycopersicum* var. M82 (tomato) and *Capsicum annuum* var. Big Dipper (pepper). In agreement with previous work on tomato and pepper heterografting (Kawaguchi et al., 2008; Andrews and Marquez, 2010), our self-grafted tomato and pepper plants exhibited 100% survival, while heterografted pepper:tomato (scion:stock notation) and tomato: pepper plants showed significantly reduced viability (75% and 37%, respectively; *P* = 8.648e-06; Supplemental Data Set S1; data collected 30 days after grafting [DAG]; Figure 1). Furthermore, in contrast to the self-grafted species, the heterografted combinations exhibited reduced foliage, asynchronous stem bulging, and the tomato:pepper grafts displayed severely stunted roots compared to the self-grafts (Figure 1, A–D). Fragility and breakage along the junction point is another classic symptom of graft incompatibility (Zarrouk et al., 2006). We performed a bend test to assess whether the biophysical integrity of the pepper and tomato heterografted junctions was significantly reduced (Movies 1 and 2). Only 6% of the self-grafted pepper stems and 0% of the self-grafted tomato stems broke at the junction, but the majority of the heterografts broke at this position (75% of pepper: tomato stems and 92% of tomato: pepper; *P* = 5.967e−11, Fisher’s exact test, Movies 3 and 4, Figure 1N; Supplemental Data Set S1).

To identify the cause of graft failure and junction fragility in the heterografts, we inspected cellular and anatomical detail of the self- and heterografted stems at 30 DAG (Figure 1, E–L; Supplemental Figure S1). Continuous xylem files span the graft junction in the self-grafted tomato and pepper plants, indicating that nutrient and water flow was restored between the scion and stock (Figure 1, E, I, H, and L). Our anatomical imaging showed that these new xylem strands formed toward the periphery of the junction, creating a thickened xylem bridge (Figure 1, E and H; Mng’omba et al., 2007). Conversely, the heterografts showed an over proliferation of disorganized metaxylem above and below the graft interface (Figure 1, F, G, J, and K). These masses of disconnected xylem files are known as anastomoses and signify a breakdown in the vascular continuity of the stem (Tiedemann, 1989). Despite fully healed epidermal and cortical layers across the junction, all of the heterografted samples failed to form vascular bridges (Figure 1, F and G). These data support a model where heterografted tomato:pepper and pepper:tomato have delayed incompatibility due to failed vascular reconnection.

### Differences between compatible versus incompatible graft anatomy form within the first week of grafting

**Figure 2:**
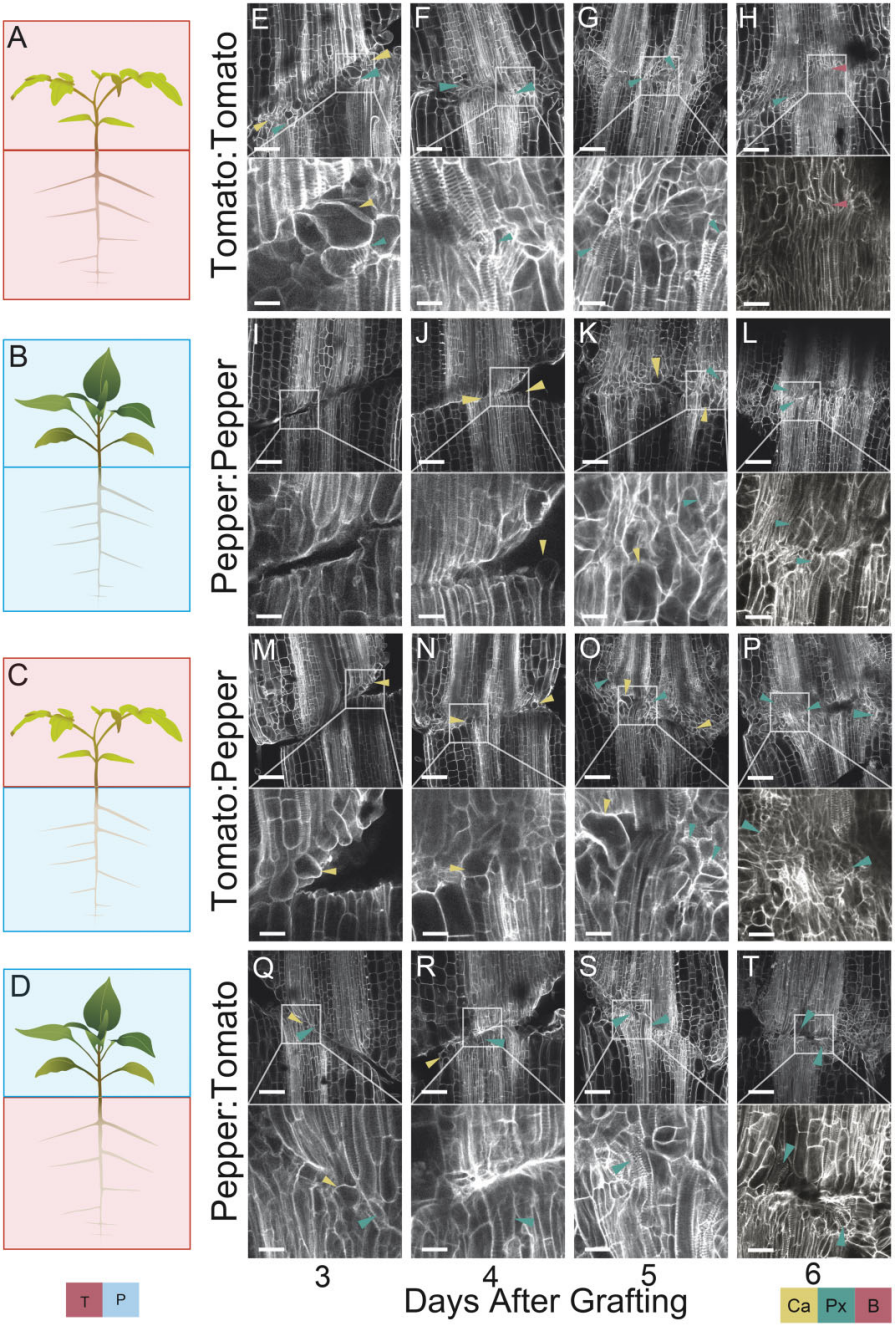
Tomato and pepper heterografts express graft incompatibility within the first week post-grafting. A–D, Graphical depictions of tomato (red boxes) and pepper (blue boxes) demonstrate self-grafted tomato (A), self-grafted pepper (B), tomato:pepper (C), and pepper:tomato (D). E–T, Anatomical timeline for self-grafted tomato (E–H), self-grafted pepper (I–L), tomato:pepper (M–P), and pepper:tomato (Q–T) collected daily from 3 to 6 DAG shows delayed vascular progression and xylem discontinuity in heterograft combinations. Newly formed callus cells are marked with yellow arrowheads, newly formed protoxylem cells are marked with blue arrowheads, and xylem bridges are marked with red arrowheads. The tissue was stained with PI and cleared in methyl salicylate. For all graft combinations and all timepoints *n* = 3. Scales bars = 200 µm, 4× inset image scale bars = 50 µm. Additional images can be found in Supplemental Figure S2.

The formation of functional vascular tissue is crucial for successful grafting. Our heterografts exhibit severe disruptions in vascular strand reconnection. To identify when these vascular phenotypes manifest, we constructed an anatomical timeline for junction formation (Figure 2), comparing self-grafted tomato (Figure 2A) and pepper (Figure 2B) with heterografted tomato:pepper (Figure 2C) and pepper:tomato (Figure 2D) junctions between 3 and 6 DAG (Figure 2, E–T). We observed parenchymatous callus formation, especially along the stem periphery in all graft combinations (Figure 2). Self-grafted tomatoes exhibited significant callus production at 3 DAG (Figure 2E), and early differentiation of bulbous callus cells into proxylem by 3–4 DAG (Figure 2F). We distinguished these transitioning callus-to-protoxylem cells based on the combination of their isometric shape and characteristic spiral cell wall thickenings (Figure 2, E and F; Esau, 1965). The vasculature continued to differentiate 5–6 DAG (Figure 2, G and H), which led to elongated xylem strands that connected across the graft junction by 6 DAG (Figure 2H).

In contrast to tomato self-grafts, self-grafted pepper stems showed significant water loss during junction formation. This, in combination with a slower rate of callus formation, increased the fragility of the pepper grafts. While pepper roughly followed the same anatomical stages as tomato, it lagged behind by about 24 h, potentially due to the increased fragility of the junction. Accordingly, we identified callus cells at 4 DAG (Figure 2J), bulbous callus-protoxylem cells at 5 DAG (Figure 2K), and early signs of vascular maturation by 6 DAG (Figure 2L).

Much like self-grafted tomato, tomato:pepper and pepper:tomato heterografts produced a considerable amount of callus along the tomato half of the junction at 3 DAG (Figure 2, M and Q). Moreover, we identified protoxylem formation between 3 and 5 DAG in both heterografts, but again, this was only on the tomato side of the junction (Figure 2, O, Q, R, and S). Thus, while in the tomato half of the heterografts exhibited parenchymatous and vascular proliferation, pepper stems remained developmentally stalled during the first 5 DAG, exhibiting no signs of protoxylem differentiation until 6 DAG (Figure 2, P and T). Pepper and tomato self-grafts exhibit mild differences (24 h) in the temporal development of the junction; however, when heterografted, pepper exhibits a strongly delayed wound response that leads to the discoordination of vascular patterning across the junction. Unlike the self-grafted plants that formed vascular connections by 6 DAG (Figure 2, H and L), heterografted plants did not form any xylem bridges across the interface, demonstrating that failed vascular connectivity manifests early in the development of this incompatible combination.

### Molecular networks support distinct hub regulators for self-grafted tomato and pepper

To identify genetic regulators that are essential for proper vascular patterning in the graft junction, we generated temporal gene regulatory networks (GRNs) for graft formation in compatible self-grafts and incompatible heterografts. Using our anatomical timeline, we selected informative sample points that are associated with crucial steps during graft formation: graft adhesion (1 DAG), callus formation (3 DAG), and protoxylem differentiation (5 DAG; Figure 2).

To generate this molecular timeline, we harvested junctions for RNA-sequencing from self-grafted and heterografted tomato and pepper combinations at 1, 3, and 5 DAG. Using pairwise comparisons amongst all three timepoints, we identified 497, 530, and 536 differentially expressed genes (DEGs: log_2_[fold change] >2 or < −2, FDR <0.05) 1, 3, and 5 DAGs in the tomato self-grafts, respectively (Supplemental Data Set S2; Supplemental Figure S3). Most DEGs are specific to one time point and only 18.12% are shared between at least two time points. Upon clustering and plotting the expression dynamics of these DEGs, we observed that only DEGs from cluster 2 show similar expression dynamics across the different graft combinations, indicating that the expression of key self-grafted DEGs is highly disrupted in the heterografts. Some clusters, for example clusters 1, 3, 4, 7, and 9, show disrupted expression dynamics in only one of the two heterografts, supporting asymmetrical regulatory disruptions.

**Figure 3:**
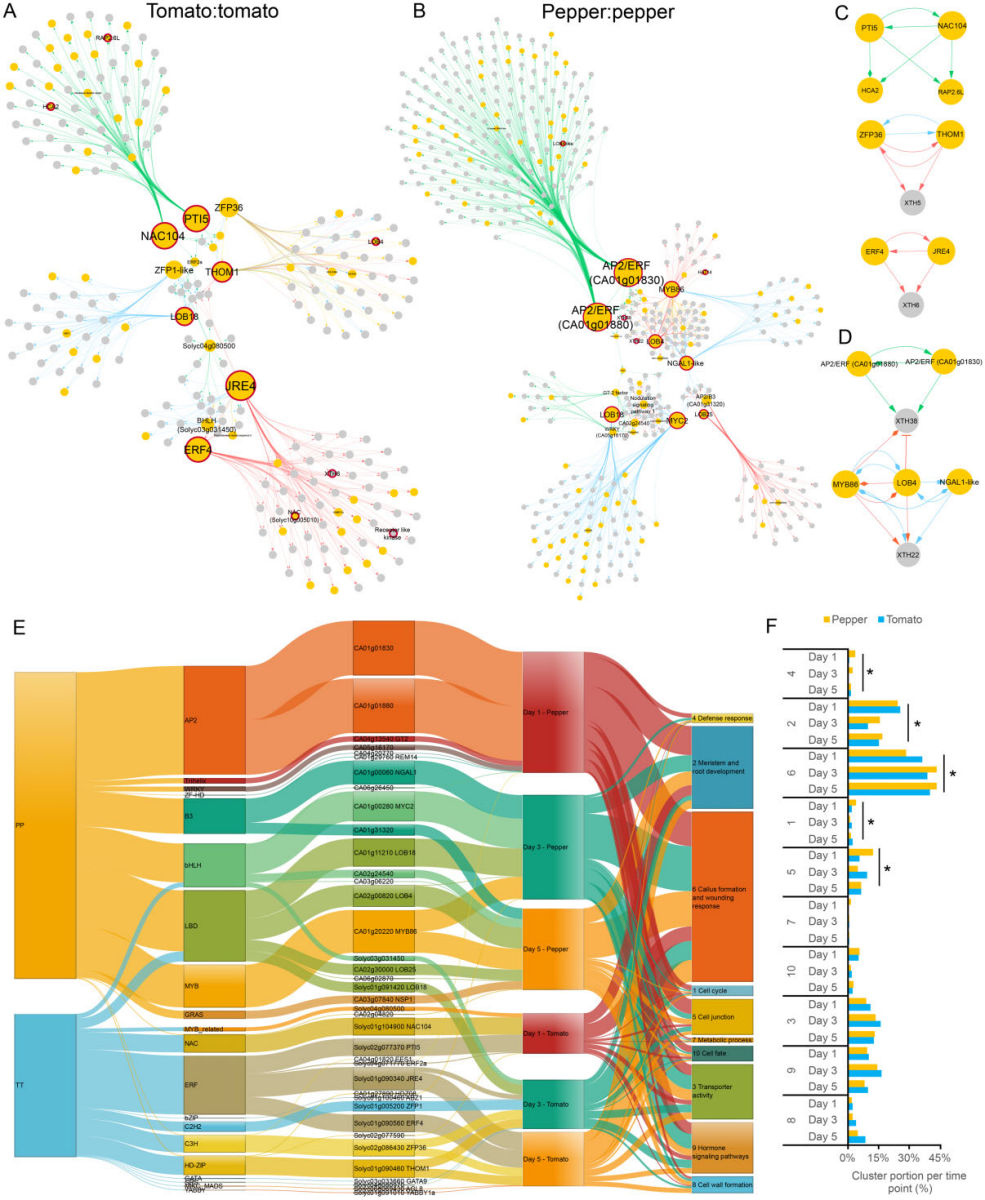
Time-specific modules and their major regulators identified in tomato:tomato and pepper:pepper self-graft gene regulatory networks. A and B, Causal relations were predicted with a dynamic Bayesian network approach between differentially expressed transcription factors and DEGs associated with GO categories related to grafting for the (A) tomato:tomato self-graft and (B) pepper:pepper self-graft. Green, blue, red, and yellow arrows represent regulations at 1 DAG, 3 DAG, 5 DAG, and 3 and 5 DAG, respectively. Yellow and gray nodes represent transcription factors and non-transcription factors, respectively. Red bordered nodes are discussed in the main text. C and D, Highlighted inferred interactions in the main text from the tomato:tomato (C) and pepper:pepper (D) networks. (E) Sankey diagram visualizing inferred gene regulatory interactions from the tomato:tomato and pepper:pepper networks. The width of the connections between each vertical block represents the number of genes (from left to right): contained within each graft combination network, within each TF family, downstream of the major hub, expressed at a specific time point, and that fall into a specific GO cluster. All TFs that have an outdegree >0 are included. F, Percentage of the downstream target genes associated with each GO cluster per time point. * = *P* < 0.05 (Fisher’s exact test).

Next, to limit the number of input genes and increase the accuracy of the GRN inference, we applied a selection method using graft-related gene ontology (GO) terms (372) critically selected based on our observations from the anatomical timeline and published studies on grafting (Figure 2; Supplemental Data Set S3 ; Melnyk et al., 2018; Xie et al., 2019). We included 168 DEGs that overlapped with the graft-related GO terms, as well as all 63 differentially expressed transcription factors (DETFs) to perform network inference (Supplemental Figure S4). Specifically, we inferred three networks for each time point with a dynamic Bayesian network approach and combined the predicted regulatory interactions, visualizing time point-specific, and common regulations (Figure 3; Supplemental Data Set S4; de Luis Balaguer et al., 2017; de Luis Balaguer and Sozzani, 2017; Spurney et al., 2020).

Within the tomato:tomato network, we identified three subnetworks or modules for each of the time points, as well as a module common for two time points (3 and 5 DAG; Figure 3A). The early time point module (1 DAG) contains 85 genes that are predominantly regulated by two TFs: an ortholog of NAC104 (Solyc01g104900), which is known to negatively regulate cell death during vascular formation, and an ERF/AP2 protein PTO INTERACTING 5 (PTI5, Solyc02g077370; Gu et al., 2002; Sari et al., 2019; Gupta et al., 2020). Within this early temporal module, we predict that NAC104 and PTI5 are controlling the expression of tomato orthologs for two *A. thaliana* genes that are functionally implicated in grafting: RELATED TO AP2 6L (*RAP2.6L*) (Solyc12g042210) and HIGH CAMBIAL ACTIVITY 2 (*HCA2*; Solyc06g071480; Figure 3C;Asahina et al., 2011; Miyashima et al., 2019).

In agreement with the anatomical observations at 3 DAG, when callus cells start to form (Figure 2A), our network predicts two major hub genes, i.e. highly connected genes, for cell proliferation: *LBD18* (Solyc01g091420) and TOMATO HOMEOBOX GENE 1 (*THOM1*) (Solyc01g090460). Notably, LBD18 functions in callus specification and maintenance, and THOM1 marks meristematic cells, both of which are crucial developmental processes during junction formation (Meissner and Theres, 1995; Ikeuchi et al., 2017). Moreover, we infer that THOM1 regulates a XYLOGLUCAN ENDOTRANSGLUCOSYLASE/HYDROLASE (*XTH*) gene (Solyc07g052980); XTHs have been shown to function in the proliferation of the pith during tissue regeneration in *A. thaliana* (Figure 3C; Pitaksaringkarn et al., 2014). Furthermore, our network infers an additional hub–XTH interaction during the late time point module (5 DAGs), where ETHYLENE RESPONSE FACTOR (ERF4; Solyc01g090560), and JASMONATE-RESPONSIVE ERF (JRE4; Solyc01g090340) co-regulate an XTH (Solyc11g065600; Figure 3C; Nakayasu et al., 2018). Overall, this analysis uncovers regulators that control downstream genes with roles in tissue regeneration and junction formation.

Our anatomical timeline for self-grafted pepper predicts delayed development of junction formation relative to self-grafted tomato. To investigate how molecular networks for graft formation are shifted between these species, we constructed a comparative GRN for pepper. We identified 1,318, 683, and 540 DEGs at 1, 3, and 5 DAG, respectively, for the self-grafted pepper data set (Supplemental Data Set S5; Supplemental Figure S5). Similar to tomato, a limited number of genes are shared between at least two time points (15.92%) and a high level of disruption was observed for the dynamic expression of these DEGs in the heterografted samples (Supplemental Figure S5). We selected graft-related DEGs and DETFs following the same criteria that we applied to self-grafted tomato gene selection for network inference (Supplemental Figure S5 and Supplemental Data Set S4; de Luis Balaguer et al., 2017; Spurney et al., 2020). This network analysis included 333 graft-related DEGs and 105 DETFs (Supplemental Figure S5; Figure 3B). Congruent with our tomato:tomato network analysis, we identified time-specific modules within the pepper:pepper network as well as TFs that are involved in regulating multiple time points. Within the early time point module (1 DAG), we identified two ERF TFs (CA01g01830, CA01g01880) as central regulators in the network (Figure 3B). This contrasts with the tomato:tomato network, where ERFs play a key role at later stages of junction formation (Figure 3A).

Furthermore, we identified MYC2 (CA01g00280, involved in jasmonate signaling), LBD18 (CA01g11210), NGATHA-LIKE 1 (NGAL1-like, CA01g00060), and MYB DOMAIN PROTEIN 86 (MYB86, CA01g20220) as major regulators of junction formation 3 DAGs (Patzlaff et al., 2003; Dombrecht et al., 2007; Soyano et al., 2008; Lee et al., 2009; Fan et al., 2012). Notably, MYB86, which has previously been associated with lignification during xylem formation (Patzlaff et al., 2003), functions as a hub at both 3 and 5 DAGs. Gene clusters that are downstream of MYB86 are associated with xylem formation, including numerous peroxidase genes, NAC-related TF genes, and *HOMEOBOX LEUCINE ZIPPER-14* (*HAT14*; Marjamaa et al., 2009; Kajala et al., 2020). We predicted additional hubs at 5 DAGs, including LBD4 (CA02g00820) and LBD25 (CA02g30000). Finally, we identified multiple interactions where hub genes (*LBD4*, *MYB86*, *NGAL1-like*, and two *ERFs* [*CA01g01880 and CA01g01830*]) converged to regulate two *XTH* genes: *XTH22* (*CA07g00520*) and *XTH38* (*CA11g08350*; Figure 3D). These hub–XTH modules are similar to the multigene regulatory modules that we found in the self-grafted tomato GRN (Figure 3C). Despite similarities in these downstream targets, we uncover distinct hub genes between our self-grafted tomato and pepper GRNs.

Next, we compared the regulations of the pepper and tomato self-grafts to (1) align the networks with our anatomical timeline, (2) identify the specific transcriptional regulations involved in the differential progression of junction formation, and (3) contrast gene networks for self-grafted pepper and tomato. To this end, we used a Sankey diagram, which allows for the comparison and visualization of the number of target genes across different samples, time points, hub TFs, and TF families (Figure 3E). To connect the target genes to the biological processes they are involved in, we grouped all 372 GO terms from those genes into 10 clusters based on semantic similarity, which represent 10 different morphological responses (Supplemental Figure S4). This approach allowed us to assess differential regulation of biological processes between tomato and pepper, and align the networks with our anatomical timeline.

In the diagram, the species, TF family, TFs with at least one downstream target, and the 10 GO clusters related to grafting, are connected based on the number of their downstream target genes. As expected, AP2/ERF TFs, which were prominent hubs in both pepper and tomato GRNs, are uncovered as key regulators for self-grafting in both species in the Sankey diagram (Figure 3E). Interestingly, the two hub AP2/ERFs in pepper are predicted to play a key role solely at 1 DAG, while tomato ERFs are major regulators at all time points (Figure 3E). Such differential identification of TF families across the time points is also observed for bHLH, LBD, NAC, C3H, and HD-ZIPs. The GO clusters with the highest gene membership at each time point include: cell cycle, meristem/root development, defense response, and cell fate at 1 DAG, transporter activity and hormone-related signaling pathways at 3 DAG, and cell wall formation at 5 DAG (Figure 3F). Although these trends are similar for both species, we observed a 15% increase in genes associated with callus formation and wounding response (Cluster 6) in self-grafted pepper between 1 and 3 DAG, while tomatoes have strong gene membership starting at 1 DAG (Figure 3E). Delayed activation of cluster 6 in pepper provides further molecular support for our anatomical timeline (Figures 2 and 3, F).

The Sankey diagram indicates that the difference in the developmental timing of junction formation between self-grafted pepper and tomato originates from the delayed induction of key TF families, such as the LBD family, and/or the absence of other key families at specific time points, for example, the AP2/ERF and NAC families. Additionally, the regulation of DEGs associated with callus formation and wounding response is delayed in the pepper self-grafts. Thus, our network analysis informs us on the molecular underpinnings for the developmental delay in pepper graft formation.

### Incompatible heterografts display severely perturbed genetic regulation

As shown in the network analysis, tomato and pepper self-grafts utilize distinct regulatory pathways to heal following grafting. Because of this, we hypothesized that the inability of tomato and pepper heterografts to form vascular connections could be due to misaligned genetic processes required for vascular differentiation across the junction. This hypothesis is further supported by the disruption of expression dynamics from the self-graft DEGs, as well as heterograft-specific DEGs in the heterografts compared to the self-grafts (Supplemental Figure S6). To fully explore the disruptions of the genetic regulation between compatible and incompatible grafts, we utilized multiple bioinformatic approaches.

First, to identify shared genetic components, we compared the graft-related DEGs from self- and heterografted plants, which uncovered 185 shared tomato genes and 401 shared pepper genes (Supplemental Figure S6 and Supplemental Data Set S5). To identify causal relationships between the DETFs and downstream graft-related DEGs in the heterograft (1,230 tomato and 1,046 pepper DEGs in PT, 416 tomato and 859 pepper DEGs in TP), we applied a regression tree with a random forest approach and generated a Sankey diagram (Supplemental Data Set S6 and Supplemental Figures S6 and S7; Clark et al., 2019). We found overlapping TF families playing a key role in both heterografts.

Within this dataset, a considerable number of orthologs for graft-related TFs from *A. thaliana* were identified, but rarely in both reciprocal graft combinations, highlighting the fact that the incompatible grafts are disrupted in genetically distinct ways (Supplemental Data Set S7). To shed light on whether the orthologs of known graft-related genes have similar roles in both species, the expression of all tomato and pepper orthologs for functionally characterized, grafting- and vasculature-related genes from *A. thaliana* (*VND6*, *VND7*, *WOX4*, COTYLEDON VASCULAR PATTERN 2 [*CVP2*], *PXY*, *HCA2*, *RAP2.6L*, ABERRANT LATERAL ROOT FORMATION 4 [*ALF4*], NAC DOMAIN CONTAINING PROTEIN 96 [*ANAC096*], and *ANAC071*) were plotted from the self-grafted and heterografted data sets. Notably, these plots show highly perturbed expression in the heterografts, compared to the self-grafts (Supplemental Figure S8; DiDonato et al., 2004; Ji et al., 2010; Sugimoto et al., 2010; Yamaguchi et al., 2010; Asahina et al., 2011; Pitaksaringkarn et al., 2014; Melnyk et al., 2018; Matsuoka et al., 2018, 2021; Smit et al., 2020). To identify additional candidates with spatially or temporally dynamic expression patterns in the heterografts, we used a modified Shannon entropy (MSE) analysis that uncovered 34 TFs, 9 of which were previously identified in graft co-expression networks (Supplemental Figures S9 and S10 and Supplemental Data Set S8; Xie et al., 2019). We honed in on the identified causal relationships between these TFs and their downstream targets that have roles in graft formation, for example we found a bHLH TF (Solyc01g098720) that regulates *ANAC071* (Supplemental Figure S10C and Supplemental Data Set S7; Asahina et al., 2011).

**Figure 4:**
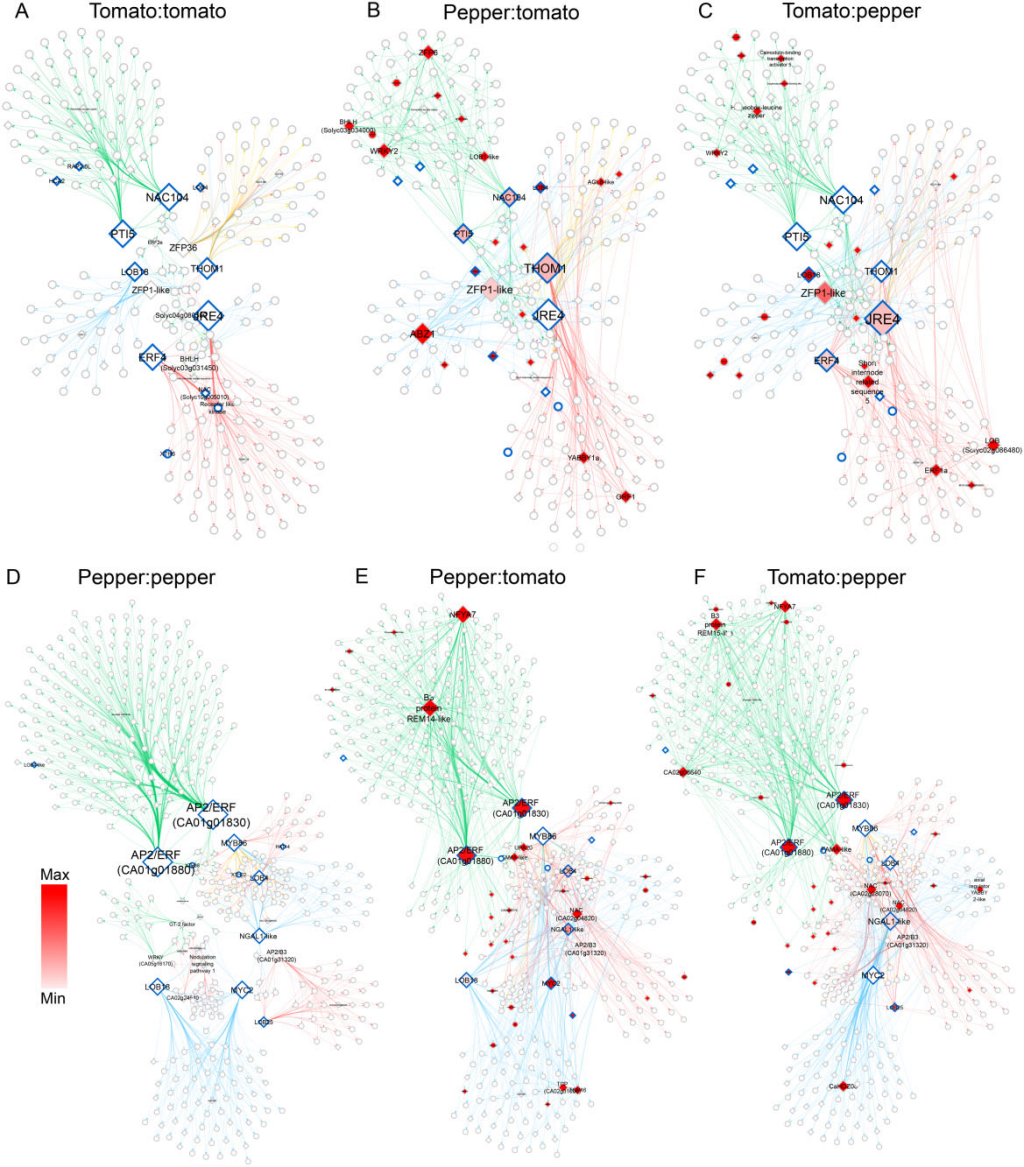
Altered and disrupted regulatory connections in incompatible heterografts. A–C, Changes in outdegree for the pepper:tomato (B) and tomato:pepper (C) networks compared to the self-grafted tomato network (A). D and E, Similarly, changes in outdegree for the pepper:tomato (E) and tomato:pepper (F) networks compared to the self-grafted pepper network (D). Green, blue, red, and yellow arrows represent regulations at 1 DAG, 3 DAG, 5 DAG, and 3 and 5 DAG, respectively. Nodes are colored with shades from white-to-red, according to the absolute magnitude of their variation in outdegree compared to the self-graft. Triangle- and circle-shaped nodes represent transcription factors and non-transcription factors, respectively. Blue bordered nodes are discussed in the main text.

To investigate how regulatory interactions from the self-grafted networks were altered during incompatible graft formation, we identified causal regulations among the self-grafted DEGs using the heterografts expression data sets with Baysian network inference and overlaid the connectivity of the newly inferred tomato:pepper and pepper:tomato heterograft networks onto our previously constructed self-graft networks (Figure 3, A and B, Figure 4; Supplemental Figure S11 and Supplemental Data Set S9). We found that many of the self-grafted hubs showed dramatic changes in outdegree (i.e. the number of outgoing edges) for the heterograft networks (Figure 4). For example, *LBD18*, a callus-related gene that acts as a central hub 3 DAG has high levels of connectivity within self-grafted tomato (outdegree = 38; Figure 4A) and pepper (outdegree = 91; Figure 4D; Supplemental Figure S11). However, we found greatly reduced connectivity for *SlLBD18* in the pepper:tomato graft (outdegree = 2; Figure 4B) and for *CaLBD18* in the tomato:pepper graft (outdegree = 2; Figure 4F; Supplemental Figure S11). Additionally, we predicted THOM1, a meristematic marker, as a major co-regulator of self-grafted tomato 3 and 5 DAG (Figure 4A); however, in the heterografts its regulatory connections have been shifted solely to 5 DAG (Figure 4, B and C). This shift in THOM1 regulation is congruent with a model where delayed specification of the vascular meristem (the cambium) is associated with disorganized vascular patterning and delayed incompatibility in the heterografts (Figures 1 and 2).

### SlWOX4 regulates xylem reconnection during graft formation

Our molecular analyses highlight shifts in gene regulatory interactions within incompatible grafts associated with wound response and callus formation; however, our anatomical observations suggest that the true culprit underlying the incompatibility between tomato and pepper relates to failed vascular reconnections. Surprisingly, few genes related to vascular formation appeared in our networks. We hypothesize that the highly DEGs within the networks likely represent calli-related regulatory pathways instead of xylem-related regulatory pathways, which is supported by our finding that DEGs associated with callus formation and wounding response are differentially regulated in tomato versus pepper.

**Figure 5:**
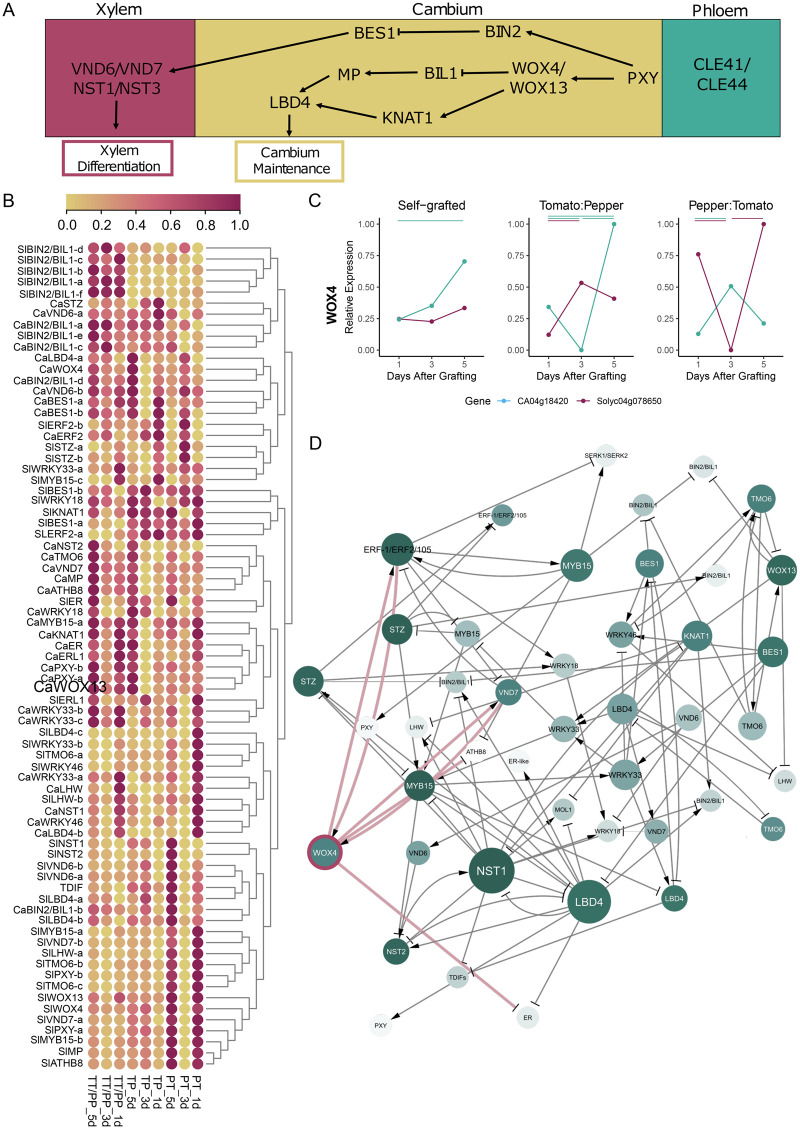
Genes involved in cambium-xylem maintenance are disrupted in heterografted plants. Schematic overview of core regulators for cambium-xylem specification (A). Scaled expression of tomato and pepper orthologs for the genes involved in cambium-xylem maintenance (B). Expression pattern of *SlWOX4* and *CaWOX4* in self-grafted and heterografted plants. Bars show significant differential expression between time points (FDR <0.05 and log_2_[fold change] >1 or < −1). Blue and red bars signify significant differential expression between pepper and tomato time points, respectively (C). Inferred regulatory interactions based on self-grafted tomato expression data for the genes included in B (D). Nodes are colored according to the magnitude of their variation in edge connections between the heterografts. Node size represents the number of outgoing interactions. WOX4 and its edges are highlighted in pink.

To investigate how cell-type population affects the downstream analyses, we analyzed the number of newly formed protoxylem in a 2D slice of the graft junction. On average, these protoxylem cells made up approximately 1% of the total area of the imaged junction (Supplemental Figure S12 and Supplemental Table S1), and thus, it is highly unlikely that genes associated with vascular formation would be pronounced in our generalized network. To connect our anatomical observations with our network predictions, we focused our subsequent analysis on tomato and pepper orthologs of *A. thaliana* genes that are involved in specifying and maintaining vascular development (Figure 5). Many of these orthologs exhibited altered expression dynamics between the compatible self-grafts and incompatible heterografts (Supplemental Figure S7). Using a regression approach, we inferred the regulatory interactions between these genes (Figure 5D).

Notably, our network inference predicts that VNDs and NSTs, which are both involved in xylem differentiation, are regulated by *WOX4* in both the self-grafts and heterografts (Figure 5C; Supplemental Figure S13). We decided to examine *SlWOX4* (Solyc04g078650) and *CaWOX4* (CA04g18420) in more detail. While these *WOX4* orthologs exhibit patterns of gradually elevated expression in self-grafted plants, their expression becomes disrupted in the heterografts (Figure 5, B and C). Notably, *WOX4* is a critical player in procambial specification and exhibits scion-dominant expression during graft junction formation in *A. thaliana* (Ji et al., 2010; Etchells et al., 2013; Melnyk et al., 2018). These results, in combination with our observation that xylem files fail to form in the incompatible heterografts, led us to the hypothesis that *WOX4* may serve a crucial function during graft formation, and disruption of this gene may lead to graft incompatibility.

**Figure 6:**
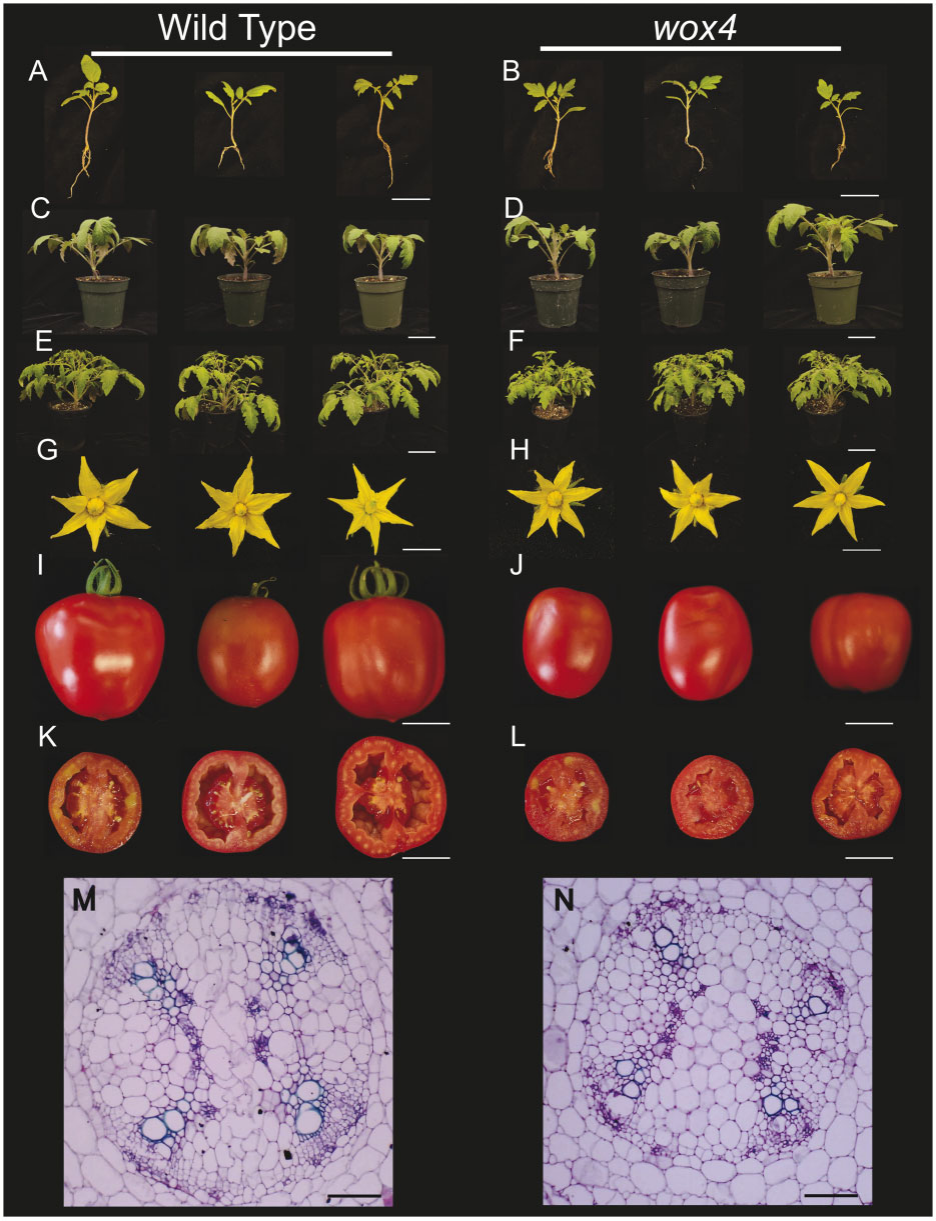
Vegetative and reproductive phenotypic characterization of *Slwox4* mutants. A–L, Representative selection of WT and *Slwox4* seedlings at the time of grafting (3 weeks after imbibition) (A and B), 5 weeks after imbibition (C and D), 8 weeks after imbibition (E and F), flowers (G and H), and fruit (I–L). M and N, Representative stem cross-sections sampled from WT (M) and *Slwox4* (N) seedlings at the developmental stage when these genotypes were grafted. Scale bar = 6 cm (A and B), 9 cm (C and D), 12 cm (E and F), 1 cm (G and H), 3 cm (I–L), and 5 mm (M and N).

To test our hypothesis that *SlWOX4* is crucial for grafting, we obtained a CRISPR–Cas9 knockout of this gene in tomato. This *Slwox4* mutant contains a 15-bp deletion in the gene coding sequence (Supplemental Figure S14). We observed that both wild-type (WT) and mutant lines had a 78% germination rate. Seedlings at the time of grafting (2 weeks old) were on average 29% smaller than WT (Figure 6; Supplemental Figure S14, A and B, Supplemental Figure S15A, and Supplemental Table S2). This size discrepancy is due to a 28% reduction in root area and 13% reduction in leaf length (Supplemental Figure S14, A–C and Supplemental Table S2). Transverse sections of the stem showed no major differences in vasculature between the two genotypes (Figure 6, M and N). Both genotypes began flowering between 7- and 8-week post-imbibition, and adult mutant and WT plants were similarly sized at 8-week post-imbibition (Figure 6, E and F). We observed no morphological differences between the mutant and WT flowers (Supplemental Figure S7, G and H); however, *Slwox4* fruit was 24% smaller than the WT (Figure 6, I–L; Supplemental Figure S15, D–H and Supplemental Table S2). This reduced fruit size can be largely attributed to a 55% reduction in locule chamber area (Figure 6, K and L; Supplemental Figure S15D and Supplemental Table S2).

**Figure 7:**
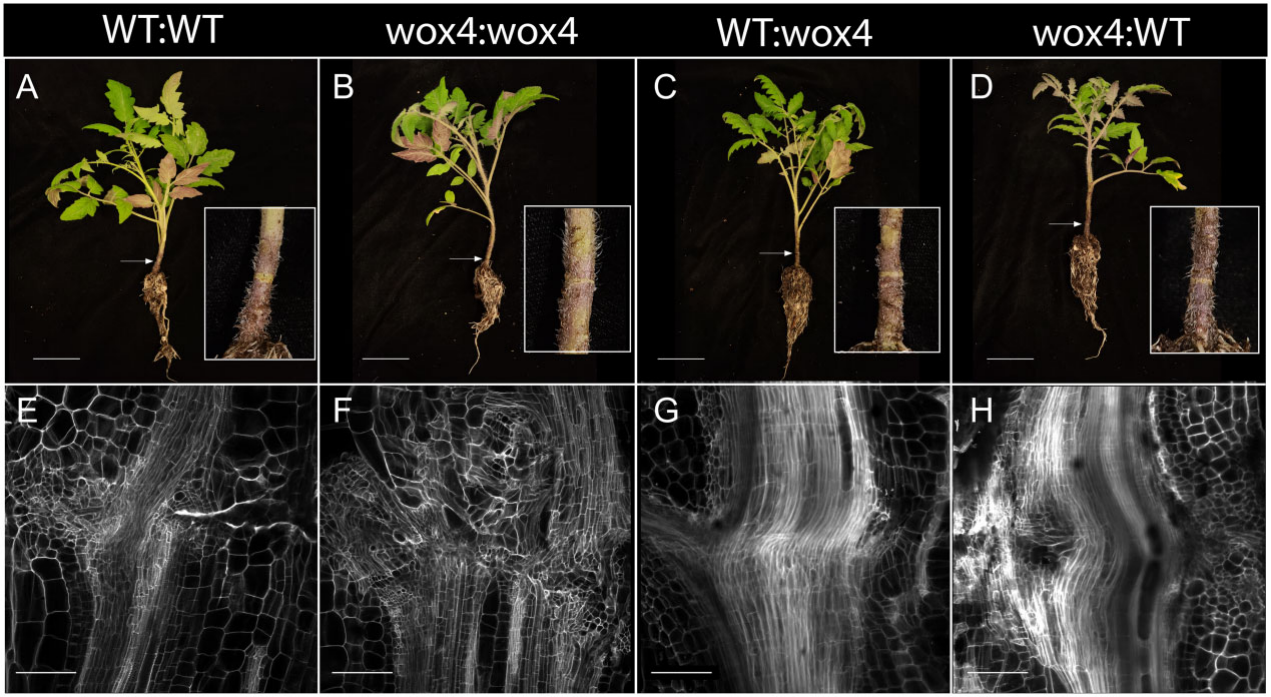
Self-grafted *Slwox4* mutants fail to form xylem bridges and thus exhibit graft incompatibility. A–H, Representative images of self-grafted WT (A and E), self-grafted *Slwox4* (B and F), WT:*Slwox4* (C and G), and *Slwox4*:WT (D and H) plants (A–D) and longitudinal sections of the graft junction (E–H) taken 30 DAG. A–D, Graft junction marked with white arrows. E–H, Tissues were stained with PI and cleared in methyl salicylate. *N* = 3, scale bars = 5 cm (A–D) and 200 μm (E–H). Additional images can be found in Supplemental Figure S17.

The connectivity of *SlWOX4* to central cambium-xylem maintenance genes indicates that this gene may be crucial for grafting, to test this, we made self- and heterograft combinations between *Slwox4* mutants and WT controls and evaluated survival as well as anatomical connectivity within the junction (Figure 7). We did not observe a statistically significant difference in the survival rate of *Slwox4* mutant versus WT grafts at 30 DAG (Supplemental Figure S16 and Supplemental Data Set S1) However, while viability was not impacted, we discovered that the self-grafted *Slwox4* junctions exhibited weak biophysical stability. Self-grafted *Slwox4* mutants were significantly more likely to break during the bend test than WT controls (85.7% and 0%, respectively; *P* = 0.004662; Supplemental Data Set S1; data collected 21 DAG; Supplemental Figure S12B). Similar to the pepper and tomato heterografts, *Slwox4* self-grafts developed over proliferating and disorganized xylem masses that failed to connect across the junction (Figure 7, B and F). In contrast, when we heterografted *Slwox4* with WT plants (i.e. in the *Slwox4*:WT and WT:*Slwox4* heterografts), the grafts formed mature xylem connections that spanned the junction and thus did not exhibit graft incompatibility (Figure 7, C, G, D, and H). Thus, we demonstrate that *WOX4* is required in at least one half of the graft junction to maintain the cambial cell population; however, there is no bias toward rootstock versus scion function. From these results, we concluded that *WOX4* plays a crucial role in xylem reconnection during junction formation.

## Discussion

The formation of a compatible graft involves the distinct anatomical processes of both nonvascular and vascular healing. While we found that both compatible and incompatible grafts achieved nonvascular healing within 1 week post-grafting, our incompatible heterografts failed to form vascular reconnections, even when examined as late as 30 days after grafting (Figures 1 and 2). However, these incompatible heterografts can survive for several months post-grafting and thus exhibit delayed incompatibility due to failed vascular coordination within the graft junction.

**Figure 8:**
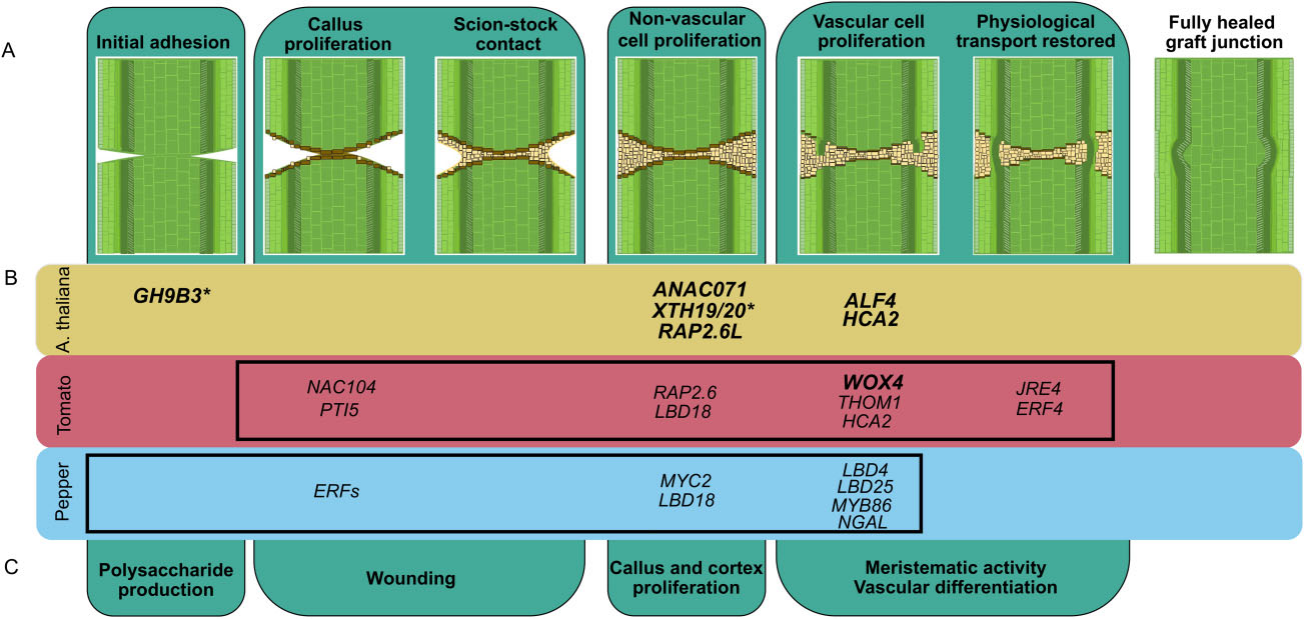
Network hubs predict new and conserved regulators for anatomical reconnection during junction formation. The anatomical timeline conserved throughout graftable plants includes initial adhesion, callus proliferation, scion–stock contact, nonvascular cell proliferation, vascular cell proliferation, and restored physiological transport through reconnected phloem and xylem strands (A). There are eight functionally characterized genes involved in graft junction formation in *A. thaliana*. We have identified 16 candidate genes for graft junction formation in tomato and pepper, many of which are described for the first time as graft-related, and one of which is the first functionally validated gene involved in vegetable crop graft formation (B). Despite the genetic diversity amongst *A. thaliana*, tomato, and pepper, all involved genes are associated with core anatomical steps along the graft junction timeline (C). The black boxes in B specify the processes captured in the anatomical timelines for tomato and pepper (Figure 2). Functionally validated genes involved in grafting are bolded. Non-transcription factors are notated with an Asterix.

Despite the widespread applications of grafting for agricultural crop improvement, only eight genes have been directly implicated in graft formation; the majority of which were discovered in *A. thaliana* (Asahina et al., 2011; Pitaksaringkarn et al., 2014; Melnyk et al., 2015, 2018; Notaguchi et al., 2020). Transcriptomic characterization of junction formation has helped elucidate both temporal and rootstock–scion-specific molecular patterns that are associated with graft formation (Asahina et al., 2011; Pitaksaringkarn et al., 2014; Melnyk et al., 2015, 2018; Xie et al., 2019; Notaguchi et al., 2020). By constructing our own anatomical timeline (Figure 2) and corresponding temporal transcriptomic data set (Figure 3), we synthesized a molecular-informed model for the developmental progression of junction formation (Figure 8). In this model, we predicted genetic hubs at 1 DAG that are associated with wound responses, including defense-related genes (*PTI5*—tomato), programmed cell death (*NAC104*—tomato), and ethylene signaling (*ERFs*—pepper). At later developmental stages, 3 and 5 DAG, we identified hubs involved in callus production (*LBD18*—tomato and pepper), meristematic activity (*THOM1*—tomato, *LBD4 and LBD25*—pepper), and hormonal signaling (*AP2/ERFs and MYC2*—pepper, *JRE4 and ERF4*—tomato). Despite genetic diversity in these regulators between tomato and pepper, we showed that our hub genes converge on the regulation of functionally related targets that are essential for grafting in *A. thaliana* (e.g. the XTH regulatory modules; Figure 3, C and D).

Previous studies have focused on understanding cell-to-cell interactions in the graft junction, with the aim of identifying graft-specific genetic factors that are independent of wound responses (Melnyk et al., 2018; Xie et al., 2019). We designed our study to investigate the involvement of wound-induced tissue regeneration during junction formation, and thus this work inherently uncovers genetic hubs that were not previously considered to play a role in grafting. These hubs have, however, been implicated in the related process of haustorium formation in parasitic plants, and thus provide molecular support for the connection between graft junctions and haustoria (Melnyk et al., 2018; Xie et al., 2019; Jhu et al., 2021). The fact that we identified diverse regulatory hubs between our species, supports a model in which grafting is not controlled by genetically conserved regulatory genes that are evolutionarily programmed into plant genomes. Rather, it is a human invention that draws on the innate capacity for plant regeneration following wounding. In this light, it is logical that the specific genetic regulators for grafting are diverse, while activation of core biological processes related to wound response and regeneration is largely conserved across species.

Because tomato/pepper heterografts fail to form coordinated xylem connections across the graft junction, we looked for orthologs of TFs that are involved in cambium-xylem maintenance in *A. thaliana* (Figure 5, A and B). We were able to identify numerous Solanaceae orthologs with disrupted expression patterns in the incompatible heterografts relative to self-grafted tomato and pepper (Figure 5B). By investigating the interconnectivity of these TFs, we identified WOX4 as a central regulator for vascular regeneration during junction formation (Figure 5D). While this role for WOX4 in grafting is logical, given its role in procambial maintenance, the translation of known vascular networks into the identification of genes that are essential for grafting has been challenging (Hirakawa et al., 2010; Ji et al., 2010; Etchells et al., 2013). Our discovery provides a new tool for disrupting graft formation at the crucial stage of xylem reconnection. Despite the apparent disruption of xylem patterning in self-grafted *Slwox4* junctions, ungrafted *Slwox4* mutants form organized vascular strands, which is likely the result of SlWOX4/SlWOX14 functional redundancy, as was previously demonstrated in *A. thaliana* (Etchells et al., 2013). Interestingly, we found that heterografted WT:*Slwox4 and Slwox4*:WT plants form mature xylem bridges across the junction, demonstrating that the requirement of *SlWOX4* expression is not direction specific. This result is somewhat surprising given previous work showing that WOX4 exhibits scion-dominant expression (Melnyk et al., 2018). Further research into the spatio-temporal patterning of *SlWOX4* during grafting will help resolve why *SlWOX4* lacks rootstock/scion biased function.

A long-standing question in the field of grafting, asks whether new vascular bridges develop through the specification of cambium or differentiate directly from callus (Crafts, 1934; Roberts, 1949; McCully, 1983; Tiedemann, 1989). Our discovery of SlWOX4 as an essential regulator of junction formation indicates that indeed, cambial specification precedes vascular differentiation. The self-grafted *Slwox4* mutants mimic incompatible graft formation, demonstrating an essential role for cambial specification in graft compatibility. Future work delving deeper into cambial patterning within the junction will help resolve how compatible grafts are determined.

## Materials and methods

### Plant materials and growth conditions

To trigger germination, *Capsicum annuum* (pepper) and *Solanum lycopersicum* (tomato) seeds were treated with 50% bleach for 30 s and then rinsed five times with sterile distilled water. Tomato seeds were germinated on wet paper towels in Phytotrays (Sigma-Aldrich) that were placed in the dark for 72 h, transferred to the light for 72 h, and then transplanted into Lambert LM-111 soil. Pepper seeds were immediately planted 1 cm deep into LM-111 soil. Tomato and pepper seedlings were grown in climate controlled chambers set to 23°C with 16:8 day/night light cycles under F54T5/841/HO fluorescent bulbs (500–800 µmol·m^-^^2^·s^-1^).

### Plant growth conditions and grafting

*Capsicum annuum* (var. Big Dipper) seeds were grown as described above. Seven days later *Solanum lycopersicum* (Var. M82) seeds were grown as described above. Twenty-one-day-old pepper seedlings and 14-day-old tomato seedlings, which have the same stem diameter, were joined with a slant or wedge graft on the internode between the cotyledons and first leaf (Kubota et al., 2008). Grafts were performed in each of the following combinations: tomato:tomato, pepper:pepper, tomato:pepper, and pepper:tomato. Grafts were held together with 1.5-mm silicon-top grafting clips (Johnny’s Selected Seeds, Albion, ME, USA). Grafted plants were generously watered, covered with plastic domes, and placed in the dark for 3 days. On day 4, plants were returned to light (500–800 µmol·m^−^^2^·s^−^^1^).

#### Graft compatibility 30 DAG

Fifty *Capsicum annuum* (var. Big Dipper) and 50 *Solanum lycopersicum* (Var. M82) seeds were grown as described above and slant grafted (Kubota et al., 2008). Plastic domes were vented 7 DAG and removed 14 DAG. Sixteen independent plants grown and grafted at the same time were collected 30 DAG. The junctions were hand-cut longitudinally, and one half was stained with propidium iodide (PI), while the other half was stained with Auramine O (details below).

#### Anatomical timeline for graft junction formation

One hundred eighty *Solanum lycopersicum* (var. M82) and 180 *Capsicum annuum* (var. Big Dipper) were grown and grafted as described above. Nine independent plants grown and grafted at the same time were collected for each graft combination, 3–6 days after grafting. Stems were fixed in formalin–alcohol–acetic acid (FAA), stained with PI, and cleared in methyl salicylate (as described below).

### CRISPR–Cas9 *Slwox4*

#### CRISPR–Cas9

CRISPR–Cas9 gRNA selection and cloning for targeting SlWOX4 was performed by the Lippman lab, as described in previous publications (Brooks et al., 2014; Soyk et al., 2019; Kwon et al., 2020). A binary vector containing two gRNAs targeting SlWOX4 (Solyc04g078650): CR-WOX4-gRNA1-TTGCAACCAAGTGTAAGTGA and CR-WOX4-gRNA2- ATCAAAAGGAGGAGTAACAA were introduced with *Agrobacterium tumefaciens*-mediated transformation into an indeterminate (Sp+) tomato cultivar M82 at the Boyce Thompson Institute Center for Plant Biotechnology Research (Van Eck et al., 2019). F2 transgenic seeds were transplanted and genotyped to confirm the absence of the Cas9 transgene and the presence of a 15-bp deletion in SlWOX4 using locus-specific primers (CR-WOX4-conf_F TGGGATCATCATCAGGAAGC and CR-WOX4-conf_R TTAGGAGGGCTATTGCTACTTTCA) as described previously (Soyk et al., 2019).

#### Mutant grafting with *Slwox4*

Indeterminate (Sp+) M82 was used for our WT control. Fifty *Slwox4* seedlings and 50 WT seedlings were grown as described above and slant grafted in the following combinations: *wox4*:*wox4*, WT:WT, *wox4*:WT, and WT:*wox4*. Plastic domes were vented 7 DAG and removed 14 DAG. Sixteen independent plants grown and grafted at the same time were collected 30 DAG. Additional images of PI stained tissue can be found in Supplemental Figure S17.

#### Phenotyping wox4

Sp+ WT and *wox4* mutants were imaged 2 weeks after germination (3 weeks after imbibition), 5 weeks after germination, 8 weeks after germination, during reproductive flowering, and fruit maturation. Stem cross sections were stained with Ruthenium Red and Toluene Blue O as described as below.

#### Protoxylem identification

Newly formed protoxylem was identified in junctions at 5 DAG. Total area was calculated by measuring 500 μm above the graft junction and 500 μm below the graft junction, representing the approximate harvest area used for RNA Seq. All stem tissue within this area was calculated in Fiji (Schindelin et al., 2012). Newly formed protoxylem cells were hand annotated and their area was calculated in Fiji. Hand annotated images can be found in Supplemental Figure S1, E, F, M, N, V, W, X, AH, AI).

### Staining and confocal imaging for graft junction anatomical analyses

#### Tissue collection

Graft junctions were harvested by cutting approximately 1 cm above and below the cut site. Tissue was placed into tissue cassettes (Sakura Finetek USA, Inc., 4117-01**)**, and immediately transferred into ice-cold FAA (10% formaldehyde, 5% acetic acid, 50% ethyl alcohol) fixative, and infiltrated under a vacuum for 2–4 h. The tissue was moved to fresh FAA and stored at 4°C overnight. The following day, tissue was moved through an ethanol dehydration series, followed by a rehydration series.

#### Propidium iodide

After fixing in FAA, and dehydrating and rehydrating tissue, the samples were stained with 20 µg·mL^−^^1^ PI (Acros Organics, CAS:25535-16-4) for 1 h and rinsed with phosphate buffered saline. Tissue was then dehydrated again in the dark, and gradually transferred into methyl salicylate clearing agent. Finally, the tissue was cleared in 100% methyl salicylate at 4°C for 2 weeks. Fully cleared graft junctions were imaged on a Zeiss LSM880 Confocal Microscope using an Argon Laser 514 nm beam.

#### Auramine O

After fixing in FAA, and dehydrating and rehydrating, tissue was stained with 0.01% Auramine O (Aldrich, CAS 861030) in 0.05-M Tris–HCl pH 7.2 for 15 min. The tissue was rinsed with water and immediately imaged on a Leica M205 fluorescent dissecting microscope using an EL6000 Mercury Metal Halide light source.

#### Ruthenium red and toluidine blue

After fixing in FAA, and dehydrating, tissue was embedded in Steedman’s Wax (Electron Microscopy Sciences, CAS 19312) as previously described (Vitha et al., 2000). Wax and tissue were oriented in molds and tissue cassettes were immediately pressed against the wax. Wax blocks were allowed to solidify overnight at room temperature. Blocks were sectioned on Leica RM 2135 rotary microtome at 10-μm thickness. Ribbons were oriented on poly-L-lysine (Electron Microscopy Sciences, CAS 63410-01) coated slides. Room temperature water was pipetted on the slides and allowed to sit for 10 min to allow for expansion. Water was then removed and slides were allowed to dry overnight at room temperature. Slides were deparaffinized in ethanol, and then hydrated. Tissue was stained for 1 min in 3% ruthenium red (Electron Microscopy Sciences, CAS 20600) and 5 min in 1% toluidine blue O (Electron Microscopy Sciences, CAS 22050; Retamales and Scharaschkin, 2014). Tissue was dehydrated and cleared in xylene. Slide covers were mounted over tissue with permount and allowed to dry overnight. Slides were imaged on a compound light microscope and images were captured with a Dino-Eye eyepiece USB camera (Model AM7025X). Three independent plants, grown at the same time, were collected, processed, and imaged for tomato (*n* = 3), pepper (*n* = 3), Sp+ WT (*n* = 3), and Slwox4 (*n* = 3), respectively.

### Bend test

Graft junction integrity was tested using manual bending. Each stem portion was held 1–2 cm away from the graft site. Even pressure was applied to bend the stem at a 45° angle. Stems that broke at the graft junction were marked as broken, stems that did not break or broke at a different point of the stem were considered not broken. Sixteen independent plants grown and grafted at the same time were tested for tomato:tomato (*n* = 16) and pepper:pepper (*n* = 16). Twelve independent plants grown and grafted at the same time were tested for tomato:pepper (*n* = 12) and pepper:tomato (*n* = 12). Eight and five independent plants grown and tested for WT:WT and Slwox4:Slwox4, respectively.

### Imaging

Grafted plants were imaged using a Samsung 12-megapixel wide-angle camera. Seedlings were imaged using Panasonic LUMIX GX85 Mirrorless Camera with a 12–32 mm lens.

### Statistical analysis of grafted plants

Statistical significance of survival and stem integrity was calculated using Fisher’s exact test. Pairwise comparisons were conducted using Fisher’s exact test. Bar plots were made in R using ggplot2 (Wickham, 2011; R Core Team, 2020). Significance was determined as *P* < 0.05. Statistical differences between WT and *wox4* plants were calculated using Student’s *t* test. Plots were made using ggplot2 and dplyr (Wickham, 2021). Aggregate plots were generated using Cowplot (Wilke, 2019).

### Construction and sequencing of RNA-seq libraries

Library construction: 50 *Solanum lycopersicum* (Var. M82) and 50 *Capsicum annuum* (var. Big Dipper) seedlings were grown as described above. Seedlings were wedge grafted. Graft junctions, consisting of 1 cm from the scion and 1 cm from the stock, were harvested between 8 and 10 PM at 1 day, 3 days, and 5 days post-grafting. Junctions were immediately flash frozen in liquid nitrogen, with one junction harvested per replicate and four independent plants per time point. RNA was extracted using TRIzol reagent (Thermo Fisher Scientific, Walthm, MA, USA). The purified RNA was treated with DNAseI (Thermo Fisher Scientific, Walthm, MA, USA), and quantified and quality checked on a DeNovix DS-11 (DeNovix, Willmington, DE, USA) spectrophotometer. RNA-seq libraries were constructed using 2.5 µg of total RNA per sample. Briefly, mRNA sequencing libraries were constructed by isolating mRNA with the NEBNext Poly(A) mRNA Magnetic Isolation Module (New England Bioloabs, Ipswich, MA, USA), followed directly by the NEBNext Ultra Directional RNA Library Prep Kit for Illumina using NEBNext Multiplex Oligos for Illumina. Six libraries were pooled per lane and run as a single-end sequencing run with 101 cycles on an Illumina HiSeq 2500 at the University of Delaware Sequencing and Genotyping Center. All sequencing data are available on GEO at https://www.ncbi.nlm.nih.gov/geo/query/acc.cgi?acc=GSE167482 (GSE167482, access token ufahgmimlzgljyb).

### Bioinformatic analyses

To analyze the time course RNAseq, reads of each sample were mapped against both tomato and pepper reference genomes with TuxNet using default settings (Supplemental Data Set S10 and Supplemental Figure S18; Spurney et al., 2020). The following reference genomes were used when running TuxNet: Pepper genome cvCM334 and Tomato genome *Solanum lycopersicum* cv Heinz (gene version ITAG3.2; Tomato Genome Consortium, 2012; Kim et al., 2014). TuxNet specifically uses the following software: Preprocessing: ea-utils fastq-mcf (Aronesty, 2013), Alignment: hisat2 (Kim et al., 2015), and differential expression analysis: Cufflinks (Trapnell et al., 2012).

Averaging across biological replicates and experimental time points, 92.59% and 82.38% reads of the tomato:tomato and pepper:pepper graft junctions were mapped to the tomato and pepper reference genomes, respectively (Supplemental Data Set S10 and Supplemental Figure S18). Mapping the heterografts resulted as expected in ∼50% alignment and a small percentage of reads (7.28% and 8.77%) mapped to the incorrect species. To increase the accuracy of the alignment of the heterograft reads, we performed a concatenation of both reference genomes, in essence, treating the heterografts as hybrids, which resulted in an 87.80% and 87.05% average alignment for the tomato:pepper and pepper:tomato heterografts, respectively.

To further explore variability, groupings, and outliers within the time course data sets, we performed a principal component analysis (PCA) that clustered the samples based on the similarity. The PCA was performed in R (version 4.0.2) using the *prcomp* function from the *stats* package. The four biological replicates clustered close together in PCs 1 and 2 of the PCA (Supplemental Figure S19). Moreover, in the PCA built using the tomato gene set, the self-grafted samples clustered together compared to the heterografts. In contrast, the PCA built with the pepper gene set showed that the samples clustered according to the time of harvest on the second principal component. Overall, the PCA verified that replicates from the same sample have similar profiles and hinted toward differences between the pepper and tomato response to grafting.

To visualize gene expression, heatmaps were generated using the scaled expression. Specifically, the expression was scaled for each gene using a min–max scaler formula, where the minimum (l) was set on 0 and the maximum (u) on 1.

The heatmap for Figure 5 was generated using Tbtools v1.064 (Chen et al., 2020), and supplemental heatmaps and plots were generated in R (version 4.0.2) using ggplot2 (Wickham, 2011).

The TuxNet interface was also used to perform gene expression analysis and infer GRNs TuxNet also includes an algorithm (TuxOP) for DEG selection using FC and FDR values. Specifically, an FDR threshold of 0.05 and log_2_ FC threshold of 2 was used. DEGs were assigned to a timepoint based on upregulation. For each data set, up- and downregulated DEGs were selected from each pairwise comparison: 1 DAG versus 3 DAG, 1 DAG versus 5 DAG, and 3 DAG versus 5 DAG, which captured all temporally regulated DEGs (Supplemental Data Sets 2, 5, and 6). To infer a GRN, DEGs associated with one of 372 manually selected GO terms (Supplemental Data Set 2) as well as all differentially expressed TFs were identified for each of the samples (Supplemental Figure S3). A total of 3,951 tomato genes and 4,375 pepper genes in the entire tomato and pepper genome, respectively, were associated with one of the 372 graft-related GO terms (Supplemental Data Set S3 and Supplemental Figure S3). To identify the GO terms associated with each gene, the GO of tomato and pepper were downloaded from dicots PLAZA 4.0 (Van Bel et al. 2018). For inferring GRNs for the self-grafts, a dynamic Bayesian network (DBN)-based inference algorithm (GENIST) was used within the TuxNet interface with a time lapse of 0 (de Luis Balaguer et al., 2017). Only putative TF-encoding genes were considered as source nodes that could regulate the expression of other DEGs. Specifically, for each time point for both self-grafts a network was generated using the selected DEGs at that time point and the average expression values from the entire time course. As such three networks were inferred for each self-graft. Finally, we combined the 1, 3, 5 DAG networks by taking the union of the three GENIST output files (Supplemental Data Set S4). The regulatory interactions between the same set of DEGs were inferred within the heterograft networks by using the average expression values from the entire time course of the heterograft for a DBN approach. Similarly, one inference was performed for each time point, after which the output of the three inference rounds were unionized in Cytoscape (Supplemental Data Set S9). The networks from the self-grafts and heterografts were compared in Cytoscape through the DyNet application (Goenawan et al., 2016). Specifically, each heterograft network was compared with the self-graft by mapping the variation of outdegree onto the node color.

For the heterograft samples, a random forest approach (RTP-STAR) within the TuxNet interface was used for network inference (Spurney et al., 2020). Similar to the self-grafts, separate networks were generated at each time point for both heterografts by using the selected DEGs and the expression values of all the replicates. Ten iterations were performed in total and the average expression values for the time course were used to determine the sign of the predicted regulations. For each of the two heterograft samples, six networks were generated: one for each time point and species genome. We combined the 1, 3, 5 DAG networks by taking the union of the three RTP-STAR output files (Supplemental Data Set S7). A total of four GRNs were generated, two for each heterograft sample, one for the tomato genes and one for the pepper genes. TuxNet is available at https://github.com/rspurney/TuxNet and video tutorials regarding installation, analysis, and network inference are freely available at https://rspurney.github.io/TuxNet/. All networks were visualized in Cytoscape 3.8.0 (Shannon et al., 2003). Sankey diagrams were generated in R with the package *networkD3* (Allaire et al., 2017; R Core Team, 2020). For the heterograft Sankey diagrams, we visualized TFs that were shown to have a major regulatory role (i.e. TFs that regulate more than 25 targets) and selected 59 TFs, which accounted for >60% of all inferred interactions.

To find orthologs across species, we used uniprot (UniProt Consortium, 2019), PANTHER (Mi et al., 2013), and generated custom orthogroupings for *Capsicum annuum*, *A. thaliana*, and *Solanum lycopersicum* using the default settings for OrthoFinder (Emms and Kelly, 2015).

Prior to comparative analyses of gene expression values, including the MSE analysis, between the self-graft aligned to their respective genomes and the heterografts aligned to the concatenated genome, the FPKM values were normalized against the self-graft 1 DAG replicate 1. To perform the semantic clustering of the 382 selected GO terms (Supplemental Figure S3 and Supplemental Data Set S3), the R package *GOSemSim* was used to compute semantic similarity (Yu et al., 2010; Yu, 2020). The computed similarity matrix was clustered into 10 clusters (optimal number of clusters identified with elbow plot from the within-clusters sum of squares) using k-means clustering.

For the MSE (Supplemental Figures S9 and S10 and Supplemental Data Set S8), first, an entropy score was calculated for each gene based on its expression values as previously described (Kadota, 2006). The lower the entropy score, the higher the variation within a genes’ expression profile. Genes with an entropy score below 30% of the max entropy were selected and outlier (1) and nonoutlier (0) scores were assigned to each of the genes’ expression values (Kadota, 2003). An outlier score of 1 indicates that the gene is upregulated for that sample compared to its entire expression profile across all samples. A nonoutlier score of 0 corresponds to where the gene is not differentially expressed. Each possible combination of outlier and nonoutlier scores (i.e. of 0 and 1) for all the expression values of each gene is generated and statistically evaluated. The statistic for evaluating outlier assignments is given by:

$$U\;=\;n\;\times \;\log(\sigma )\;+\;\sqrt{2}\;\times s\;\times \frac{\log(n!)}{n}$$

where s is the number of outlier candidates, n is the number of nonoutlier candidates, and σ is the standard deviation of the expression values of the nonoutlier candidates. The best combination of outlier and nonoutlier scores (i.e. of 0 and 1) is the one that achieves the lowest U value. Outlier scoring enables high-resolution combinatorial DEG selection via selection of genes that are labeled as outliers or nonoutliers in specific user-chosen samples. R-code used to perform the MSE is available at https://github.com/LisaVdB/MSE.

### Accession numbers

NAC104 (Solyc01g104900), PTI5 (Solyc02g077370), RAP2.6L (Solyc12g042210), HCA2 (Solyc06g071480), LBD18 (Solyc01g091420), THOM1 (Solyc01g090460), XTH (Solyc01g090460), XTH (Solyc11g065600), ERF4 (Solyc01g090560), JRE4 (Solyc01g090340), ERF (CA01g01830), ERF (CA01g01880), MYC2 (CA01g00280), LBD18 (CA01g11210), NGAL1-like (CA01g00060), MYB86 (CA01g20220), HD-ZIP14 (CA10g19210), LBD4 (CA02g00820), LBD25 (CA02g30000), XTH22 (CA07g00520), XTH38 (CA11g08350), WOX4 (Solyc04g078650), WOX4 (CA04g18420), NST2 (CA01g34750), LBD4 (CA02g00820), BIN2/BIL1 (CA02g13610), WOX14 (CA02g19960), BIN2/BIL1 (CA02g29760), VND6 (CA03g15410), PXY (CA03g15770), PXY (CA03g15770), ER-like (CA03g17140), BIN2/BIL1 (CA04g09860), SERK1/SERK2 (CA04g14890), KNAT1 (CA04g16610), STZ (CA04g17920), WOX4 (CA04g18420), BES1 (CA04g20150), MP (CA04g23050), ATHB8 (CA04g23050), PXY (CA05g14410), PXY (CA05g14410), ERF-1/ERF2/105 (CA05g14470), VND6 (CA06g06410), MYB15 (CA06g10340), WRKY33 (CA06g13580), VND7 (CA06g14050), TMO6 (CA06g23590), MOL1 (CA07g02560), BIN2/BIL1 (CA07g14640), ER (CA08g03060), WRKY46 (CA08g08240), WRKY33 (CA09g11940), WRKY33 (CA09g11950), NST1 (CA11g08290), LHW (CA11g16160), BES1 (CA12g17430), LBD4 (CA12g22480), WRKY46 (Solyc01g095630), BIN2/BIL1 (Solyc01g101000), LBD4 (Solyc02g069440), BIN2/BIL1 (Solyc02g072300), WOX14 (Solyc02g082670), BIN2/BIL1 (Solyc02g086670), MYB15 (Solyc03g005570), BIN2/BIL1 (Solyc03g006070), ER-like (Solyc03g007050), VND6 (Solyc03g083880), PXY (Solyc03g093330), ERF-1/ERF2/105 (Solyc03g093610), SERK1/SERK2 (Solyc04g072570), KNAT1 (Solyc04g077210), STZ (Solyc04g077980), WOX4 (Solyc04g078650), BES1 (Solyc04g079980), MP (Solyc04g081235), ATHB8 (Solyc04g081235), PXY (Solyc05g051640), ERF-1/ERF2/105 (Solyc05g052050), TMO6 (Solyc06g005130), VND6 (Solyc06g034340), MYB15 (Solyc06g053610), VND7 (Solyc06g065410), WRKY33 (Solyc06g066370), LHW (Solyc06g074110), TMO6 (Solyc06g075370), MOL1 (Solyc07g005010), BIN2/BIL1 (Solyc07g055200), NST2 (Solyc08g008660), ER (Solyc08g061560), WRKY18 (Solyc08g067340), WRKY18 (Solyc08g067360), WRKY33 (Solyc09g014990), TDIFs (Solyc09g061410), MYB15 (Solyc09g090130), NST1 (Solyc10g005010), VND7 (Solyc11g018660), LBD4 (Solyc11g045530), LHW (Solyc11g068960), TMO6 (Solyc11g072500), BIN2/BIL1 (Solyc12g062870), STZ (Solyc12g088390), BES1 (Solyc12g089040), LBD4 (Solyc12g100150).

## Supplemental data 

The following materials are available in the online version of this article.

**Supplemental Figure S1**. Despite vascular similarity at time of grafting tomato and pepper fail to form vascular bridges 30 days after grafting (DAG) (supports Figure 1).

**Supplemental Figure S2**. Self-grafted tomato and pepper, heterografted tomato and pepper plants 3 to 6 days after grafting (DAG) (supports Figure 2).

**Supplemental Figure S3**. Dynamic expression patterns of differentially expressed genes (DEGs) are highly disrupted in the heterografts compared to the self-grafts (supports Figures 3 and 4).

**Supplemental Figure S4**. Clustering of the 372 selected GO terms based on semantic similarity (supports Figure 3).

**Supplemental Figure S5**. Selection of differentially expressed genes (DEGs) associated with grafting for network inference (supports Figures 3, 4 and Supplemental Figure S7).

**Supplemental Figure S6**. Common differentially expressed genes (DEGs) between the self-grafts and heterografts (supports Figure 3).

**Supplemental Figure S7**. Sankey diagram visualizing inferred gene regulatory interactions from the tomato:pepper and pepper:tomato networks (supports Figure 4).

**Supplemental Figure S8**. Graft-specific genes from *A. thaliana* are disrupted during tomato and pepper heterografting (supports Figure 4).

**Supplemental Figure S9**. Heatmap of MSE-selected genes (supports Figure 4).

**Supplemental Figure S10**. Expression pattern of 37 selected transcription factors (supports Figure 4 and Supplemental Figure 9).

**Supplemental Figure S11**. Variation in outdegree in the self-grafts and heterografts (supports Figure 4).

**Supplemental Figure S12**. Protoxylem cells make up a minority of the newly formed tissue in the graft junction (supports Figure 5).

**Supplemental Figure S13**. WOX4 regulates xylem differentiation genes, *VND6/7* and *NST1/2*, in self-grafts and heterografts (supports Figure 5).

**Supplemental Figure S14**. *Slwox4* mutants exhibit minor alterations in vegetative phenotypes (supports Figure 6).

**Supplemental Figure S15**. Quantitative analysis of *Slwox4* vegetative and reproductive phenotypes (supports Figure 6).

**Supplemental Figure S16**. *Slwox4* mutant seedlings do not display decreased viability 30 days after grafting (DAG) (supports Figure 7).

**Supplemental Figure S17**. Self-grafted *Slwox4* fail to form xylem bridges and thus exhibit graft incompatibility (supports Figure 7).

**Supplemental Figure S18**. Concatenated genome improves read alignment percentage for heterografted pepper and tomato (supports Figure 3).

**Supplemental Figure S19**. Principle component analysis (PCA) of the RNAseq samples (supports Figure 3).

**Supplemental Table S1**. Protoxylem area calculation 5 days after grafting (DAG).

**Supplemental Table S2**. Phenotypic measurements of wild type (WT) and *Slwox4* vegetative and reproductive organs.

**Supplemental Data Set S1**. Statistical analysis of count data from survival of self- and heterografted tomato and pepper (red), break tests from self- and heterografted tomato and pepper (blue), survival of self- and heterografted WT and *Slwox4* (yellow) and break tests from self-grafted WT and self-grafted *Slwox4* (gray).

**Supplemental Data Set S2**. Gene expression values in tomato:tomato self-graft.

**Supplemental Data Set S3**. Three hundred seventy-two GO terms critically selected based on our observations from the anatomical timeline and published studies on grafting that were used in the generation of our GRNs.

**Supplemental Data Set S4**. Output of the network inference algorithm, dynamic Bayesian modeling, performed with the GENIST of the TuxNet software.

**Supplemental Data** **Set S5**. Gene expression values in pepper:pepper self-graft.

**Supplemental Data** **Set S6**. Gene expression values in tomato:pepper and pepper:tomato heterografts.

**Supplemental Data** **Set S7**. Output of the network inference algorithm, regression tree analysis, performed with the RTP-STAR of the TuxNet software.

**Supplemental Data** **Set S8**. MSE analysis.

**Supplemental Data** **Set S9**. Output of the network inference algorithm, dynamic Bayesian modeling, performed with the GENIST of the TuxNet software.

**Supplemental Data** **Set S10**. Reads alignment percentages of self- and heterografted samples mapped to the tomato, pepper, or concatenated (conc) reference genome.

## Supplementary Material

koab246_Supplementary_Data

## Notes

H.R.T, M.H.F., L.V.d.B., and R.S. conceived and designed the study. H.R.T. and M.H.F. gathered experimental data. H.R.T. and L.V.d.B analyzed the data. R.J.S. analyzed the RNAseq data. L.V.d.B. performed the bioinformatics analyses. H.R.T., M.H.F., L.V.d.B., R.J.S, and R.S. wrote the manuscript.

The author responsible for distribution of materials integral to the findings presented in this article in accordance with the policy described in the Instructions for Authors (https://academic.oup.com/plcell) is: Margaret Frank (mhf47@cornell.edu).
