# Supplementary material for: Gene regulatory networks for compatible versus incompatible grafts identify a role for SlWOX4 during junction formation
Source: Plant Cell. 2021 Oct 5;34(1):535–56. doi: 10.1093/plcell/koab246 (PMC8846177; doi:10.1093/plcell/koab246)
Supplement: koab246_Supplementary_Data [file koab246_Supplementary_Data.zip › tpc.21.00627_SupplementalFiguresandTables.pdf]

Supplemental Data. Thomas et al. (2021). Plant Cell

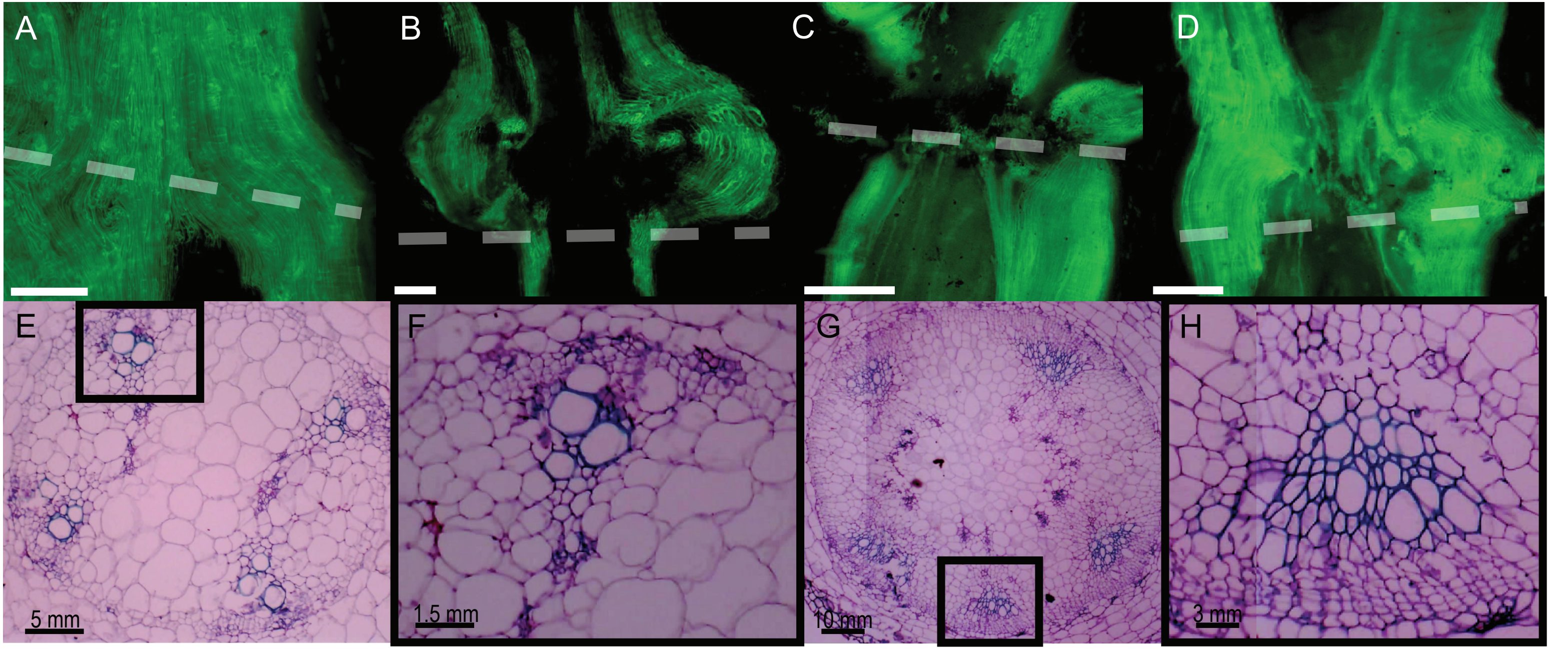

Supplemental Figure S1 Despite vascular similarity at time of grafting tomato and pepper fail to form vascular bridges 30 days after grafting (DAG) (Supports Figure 1). (A-D) Representative fluorescent images through hand sections of graft junctions showing xylem bridge formation of self-grafted tomato (A), heterografted tomato:pepper (B) pepper:tomato (C), and self-grafted pepper (D) harvested 30-days after grafting. Lignified cells were stained with Auramine O to xylem display profiles. Dashed lines indicate the original graft site. Representative images of tomato (E,F) and pepper (G,H) stems at graft site. A close up of a vascular bundle is shown for tomato (F) and pepper (H). Tissue was formalin fixed and wax embedded, sectioned, and stained with ruthenium red and toluidine blue. Tomato n = 3, Pepper n = 3. Scale bar = 5 mm (A), 10 mm (B), 1.5 mm (C), 3 mm (D).

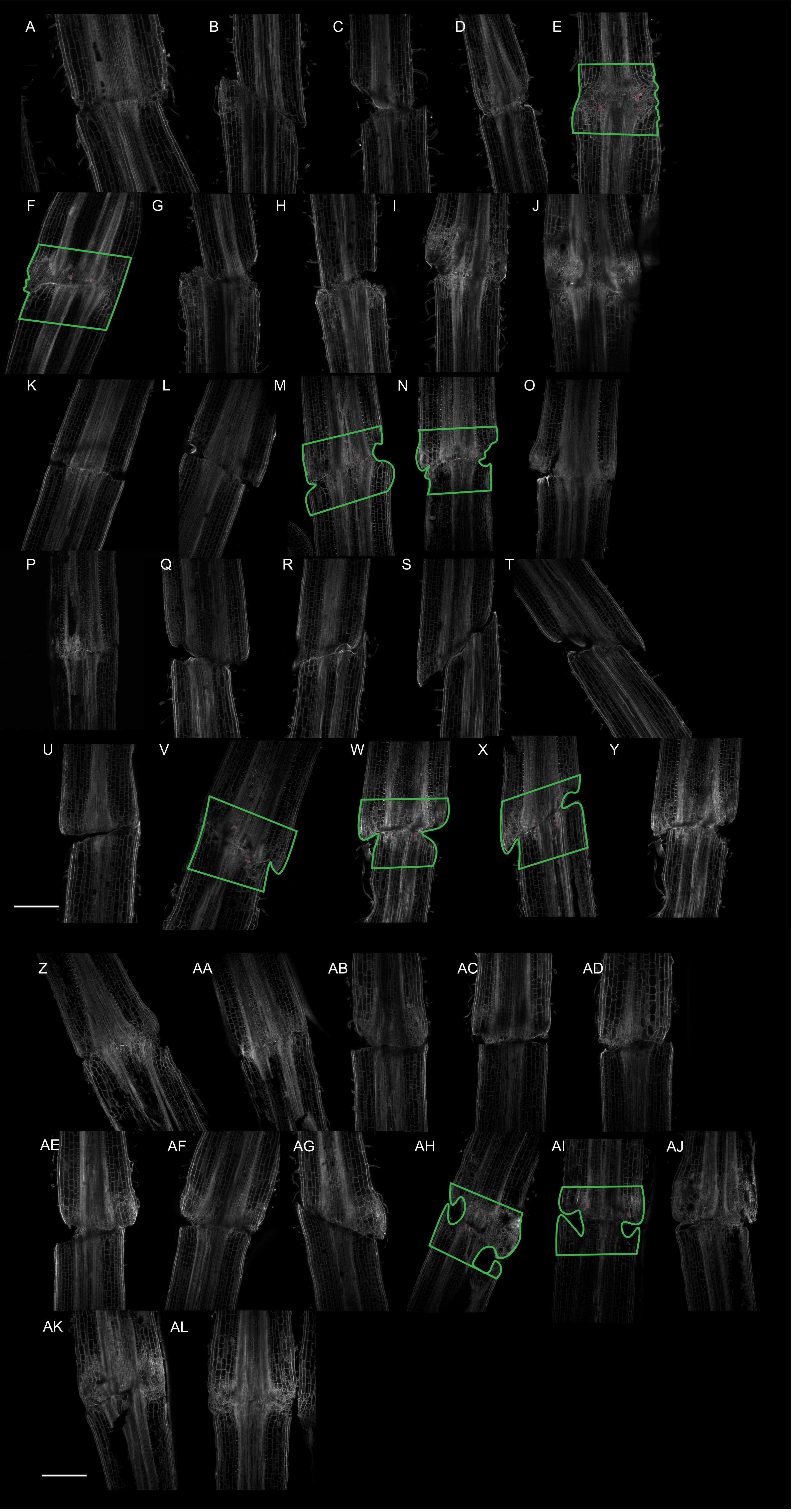

Supplemental Figure S2 Self-grafted tomato and pepper, heterografted tomato and pepper plants 3 to 6 days after grafting (DAG) (Supports Figure 2). (A-J) All images of self-graft tomato 3 DAG (A-B), 4 DAG (C-D), 5 DAG (E-F), and 6 DAG (G-J). (K-P) All images of self-grafted pepper 3 DAG (K-L), 4 DAG (M-N), and 5 DAG (O-P). (Q-Y) All images of heterografted pepper:tomato 3 DAG (Q-R), 4 DAG (S-T), 5 DAG (U-W), and 6 DAG (X-Y). (Z-AL) All images of heterografted tomato:pepper 3 DAG (Z-AA), 4 DAG (AB-AE), 5 DAG (AF-AG), and 6 DAG (AH-AL). Figures E, F, M, N, V, W, X, AH, and AI show the newly formed protoxylem cells 5 DAG and are highlighted with green contours. Scale bar = 800 µm (A-AL).

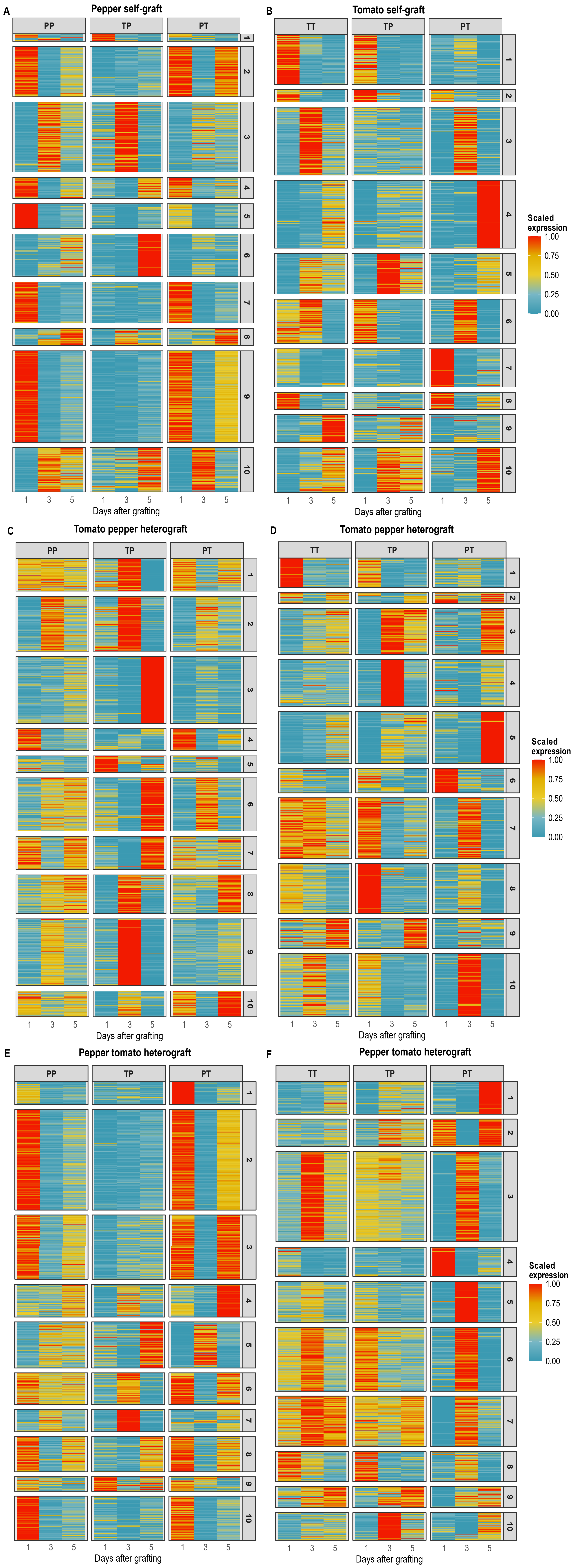

Supplemental Figure S3 Dynamic expression patterns of differentially expressed genes (DEGs) are highly disrupted in the heterografts compared to the self-grafts. (A-F) The expression values of DEGs (FDR < 0.05 and $\log_2$ fold change > 2 or < -2) identified in the PP (A), TT (B), TP (C - pepper genes, D - tomato genes), and PT (E - pepper genes, F - tomato genes) were scaled between 0 (blue) and 1 (red) and clustered into 10 clusters.

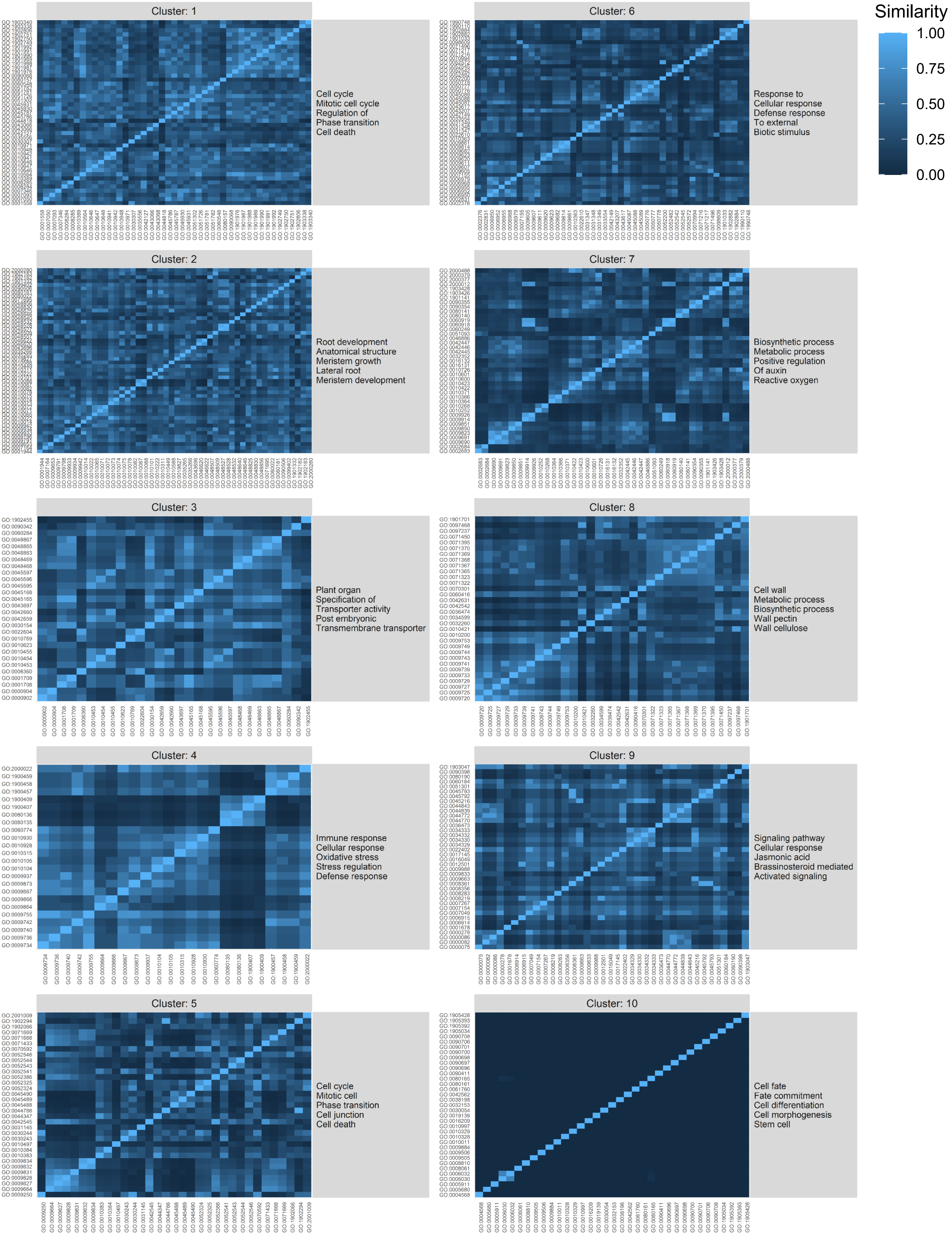

Supplemental Figure S4 Clustering of the 372 selected GO terms based on semantic similarity (Supports Figure 3). A total of 10 GO clusters were identified that include different biological processes related to each other. The percent of overlapping gene membership across GO terms is visualized on a color scale from navy-to-light blue, representing 0-100% overlap.

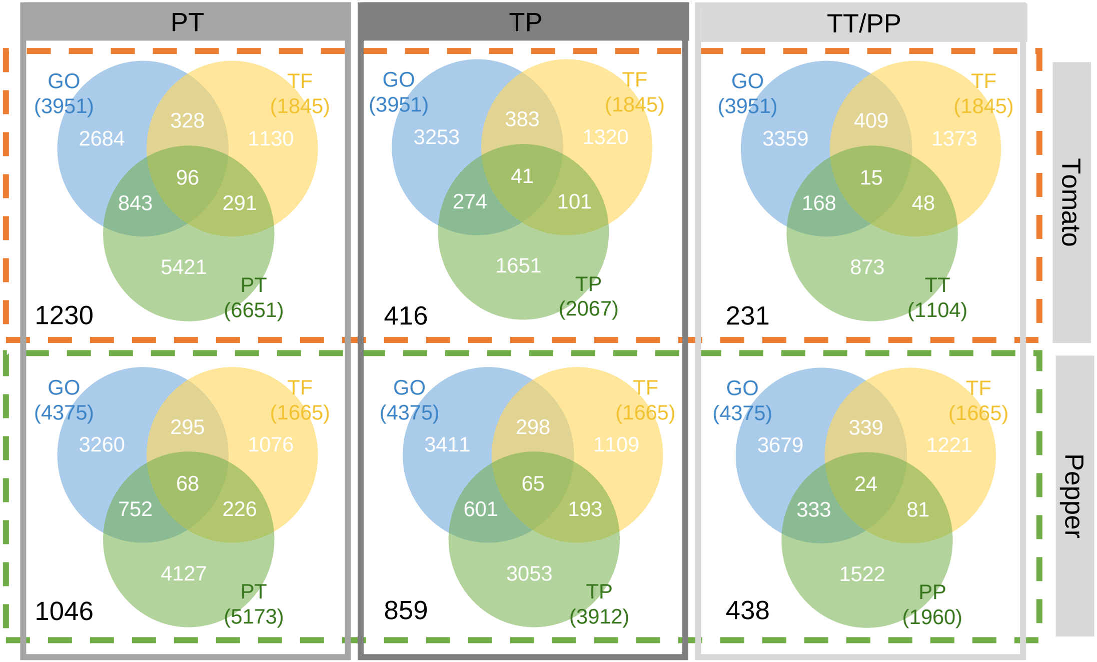

Supplemental Figure S5 Selection of differentially expressed genes (DEGs) associated with grafting for network inference (Supports Figure 3, 4 and Supplemental Figure S7). For each tissue and species, three sets of genes were compared with Venn diagrams: 1) DEGs from the RNAseq analysis, 2) all known transcription factors for tomato and pepper, and 3) genes related to the 372 selected GO-terms. The number of genes within the overlap between set 1 and 2 and the set 2 and 3 are listed in the left bottom corner of each tissue/species combination and are used for network inference.

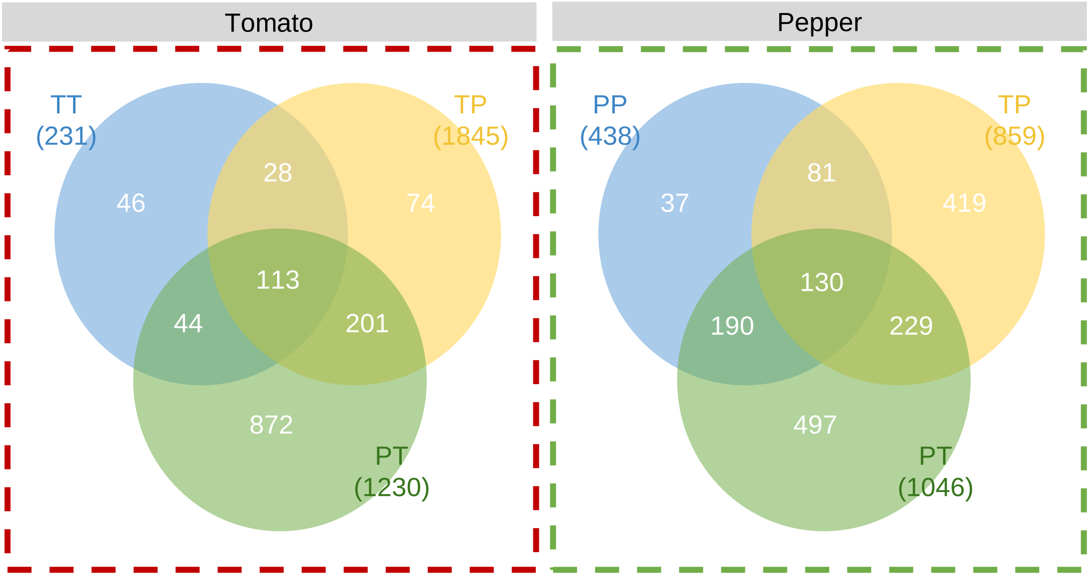

Supplemental Figure S6 Common differentially expressed genes (DEGs) between the self grafts and heterografts (Supports Figure 3). The 185 and 401 overlapping between self graft and heterografts in tomato and pepper correspond to 80% and 92% of the total DEGs of the self grafts and to 14% and 26% of the total DEGs of the heterografts, respectively. Genes from the self-graft and heterograft datasets were selected based on the overlap between temporal DEGs and our selected GO categories.

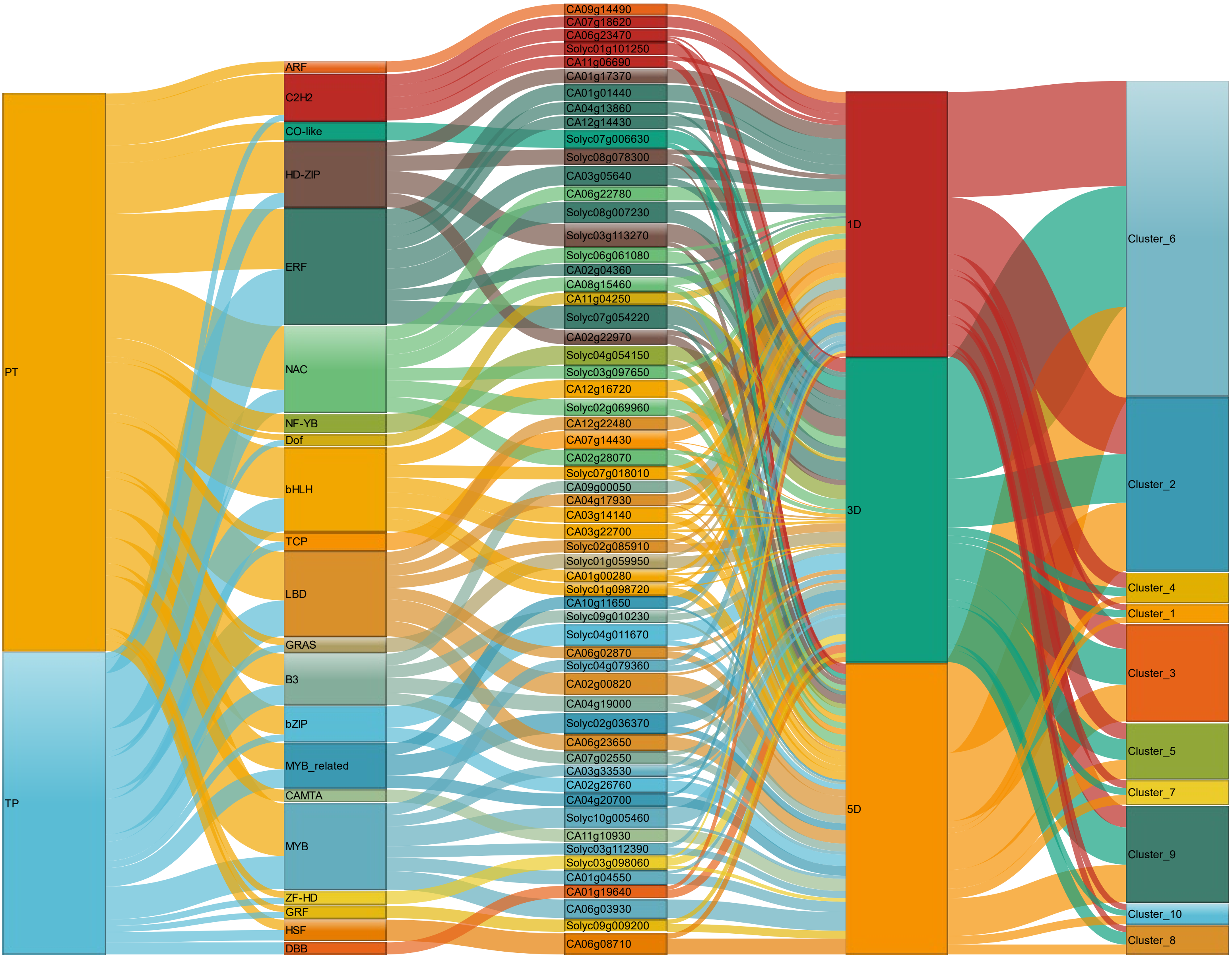

Supplemental Figure S7 Sankey diagram visualizing inferred gene regulatory interactions from the tomato:pepper networks. All TFs that have an outdegree > 25 are included. The width of the connections between each vertical block represent the number of target genes that are predicted to be directly downstream of a TF (third vertical block), to be downstream of a TF family (second vertical block), to be regulated within a tissue (first vertical block), or upon a specific time point (fourth vertical block).

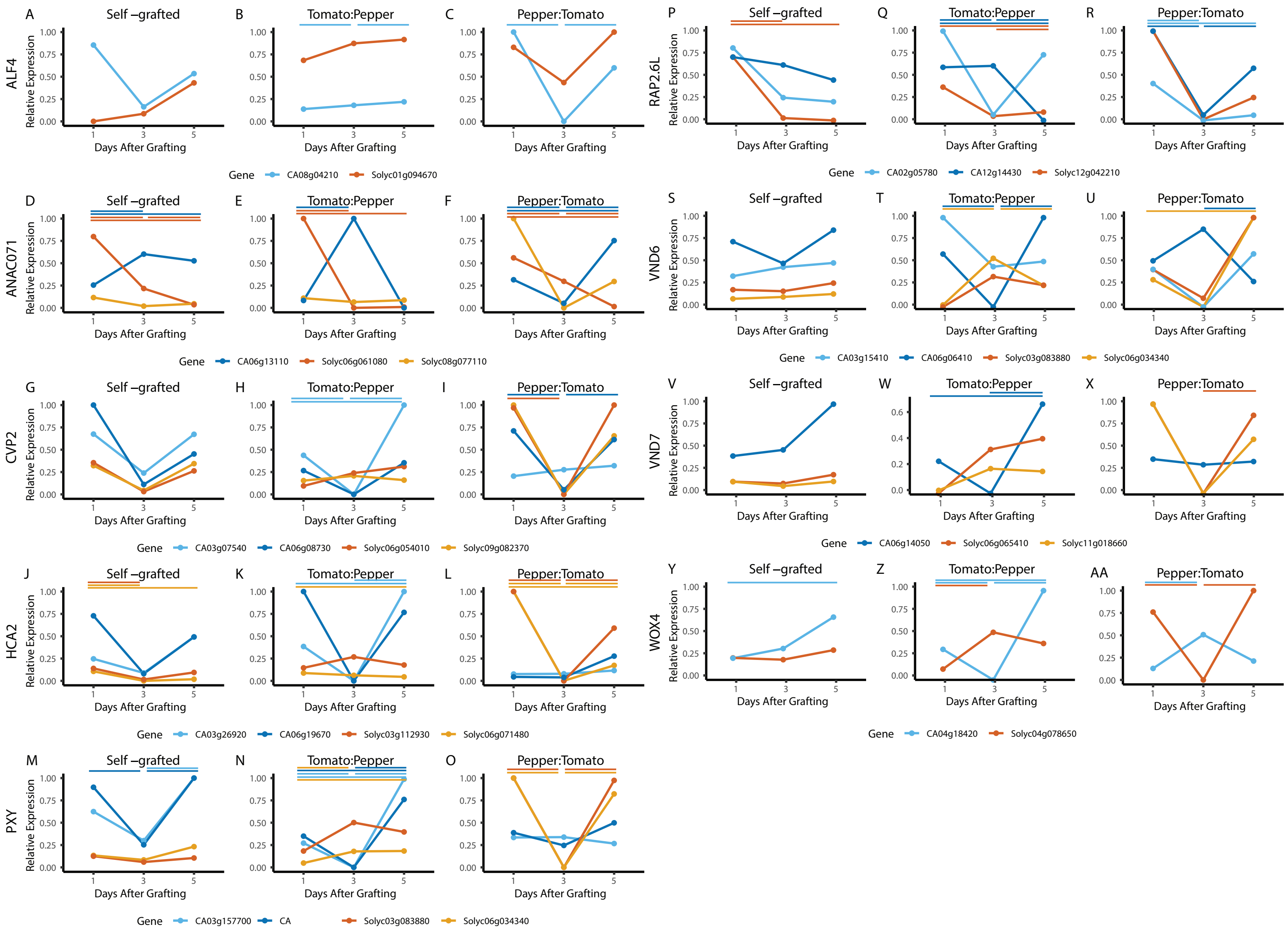

Supplemental Figure S8 Graft-specific genes from arabidopsis are disrupted during tomato and pepper hetereografting (Supports Figure 4). Tomato and pepper orthologs of known genes required for grafting (*ALF4* [A-C], *ANC071/ANAC096* [D-F], *HCA2* [J-L], *RAP2.6L* [P-R]), vascular cambium patterning (*PXY* [M-O], *WOX4* [Y-AA]), protophloem patterning (*CVP2* [G-I]), and proto- and metaxylem patterning (*VND6* [S-U], *VND7* [V-X]) shown over time in all graft combinations. Expression was normalized across all three genomes and scaled between 0 and 1. Bars show significant differential expression between time points (FDR < 0.05 and $\log_2$ fold change > 1 or < -1). Blue and red bars signify significant differential expression between pepper and tomato time points, respectively.

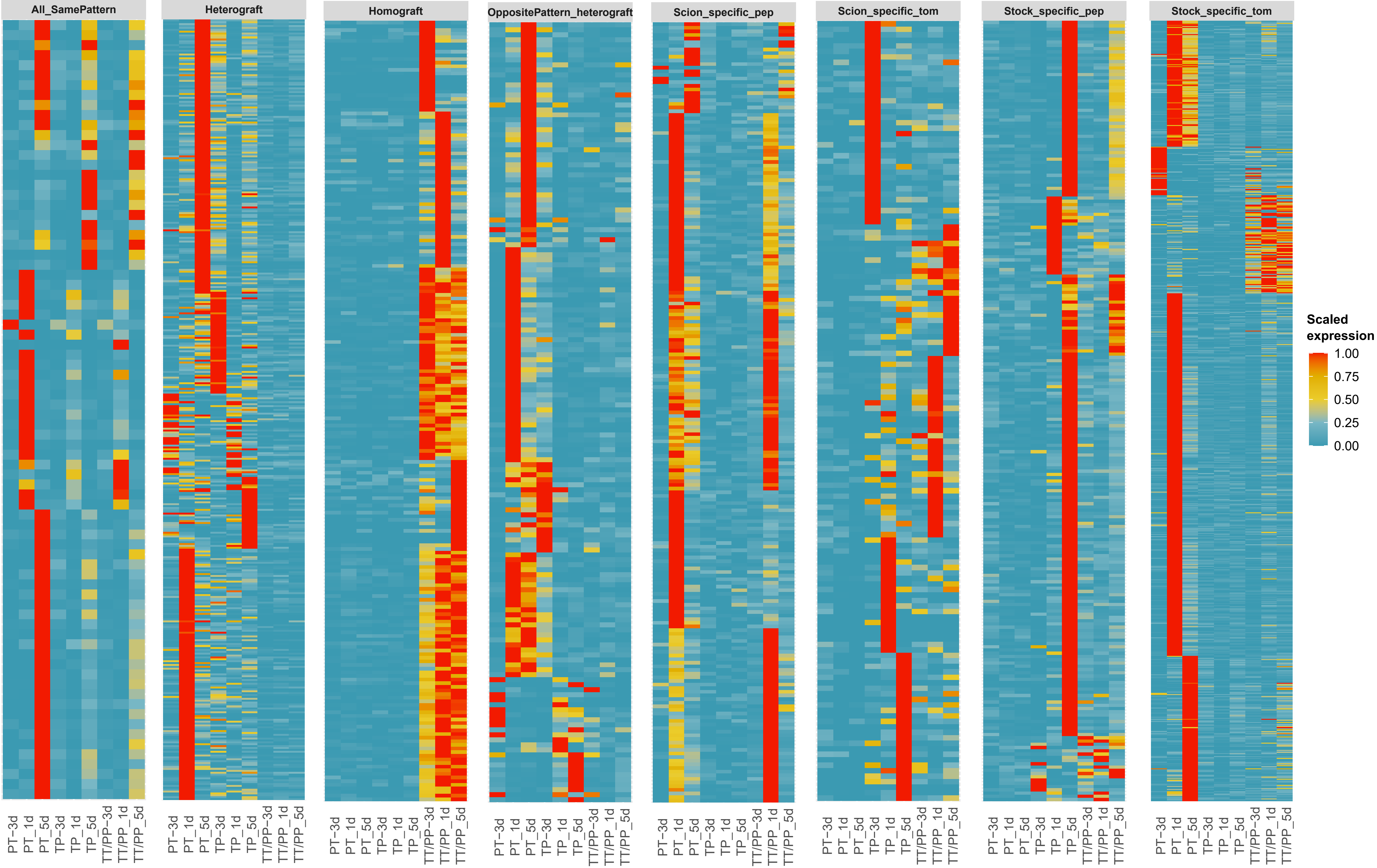

Supplemental Figure S9 Heatmap of MSE-selected genes (Support Figure 4). The heatmap shows the min/max rescaled FPKM values of the 2215 genes selected with modified Shannon entropy (MSE). These 2215 are further subdivided based on their expression pattern in eight groups (from left to right): genes that showed the same dynamical pattern across the self-grafts and heterografts (All_SamePattern), genes specifically induced in the heterograft samples (Heterograft), genes specifically induced in the self-grafts (Homograft), genes that showed an opposite dynamical pattern in the two heterografts (OppositePattern_heterograft), genes specifically induced in the scion of pepper (Scion_specific_pep), genes specifically induced in the scion of tomato (Scion_specific_tom), genes specifically induced in the stock of pepper (Stock_specific_pep), genes specifically induced in the stock of tomato (Stock_specific_tom).

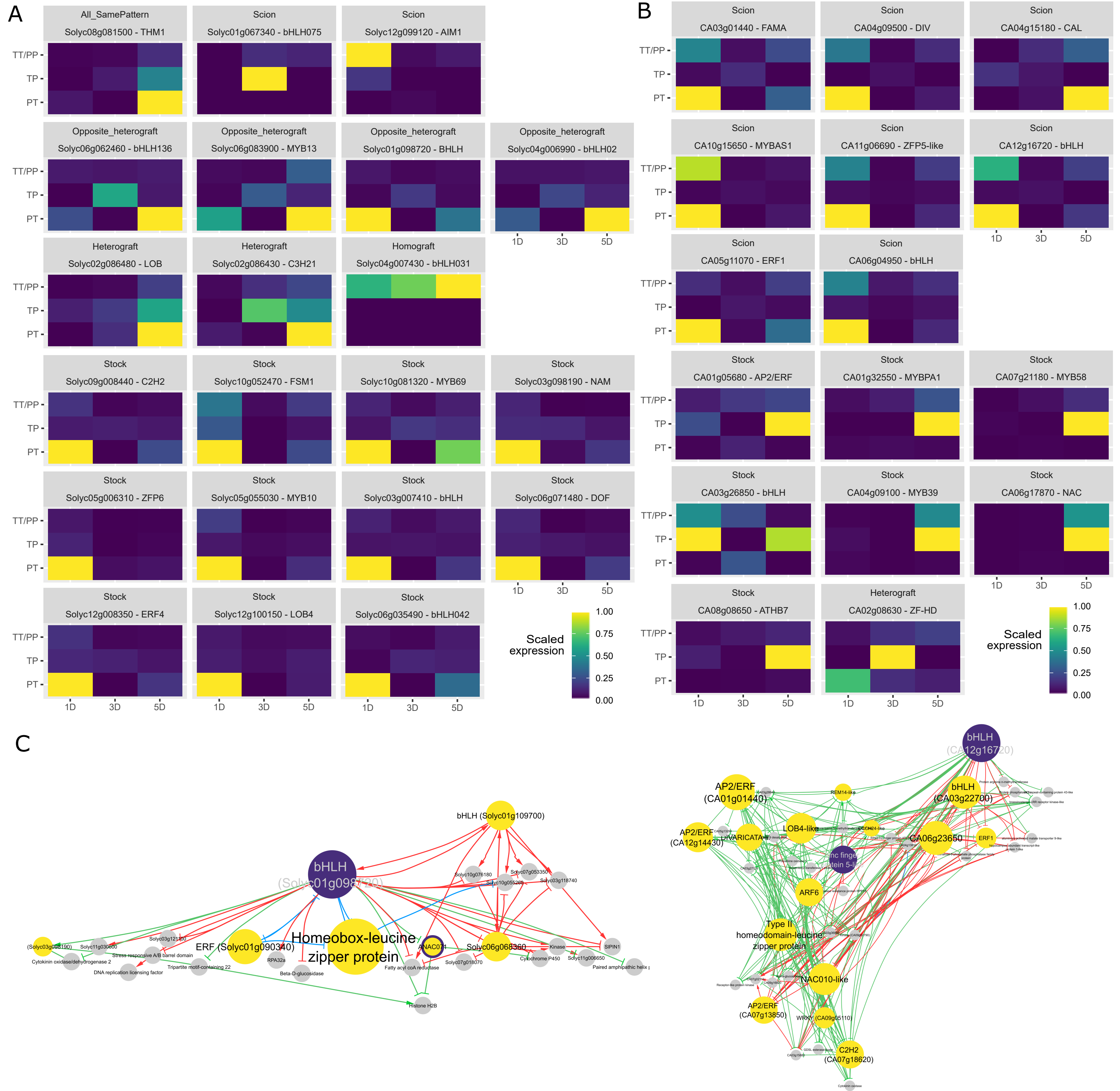

Supplemental Figure S10 Expression pattern of 37 selected TFs (Supports Figure 4, Supplemental Figure S9). (A-B) The rescaled expression values of 37 tomato (A) or pepper (B) TFs in common between the modified Shannon entropy (MSE) analysis (Supplemental Dataset S7) and GRN inference (Supplemental Dataset S6) is shown. These TFs belong to one of eight groups genes that showed the same dynamical pattern across the self-grafts and heterografts (All_SamePattern), genes that showed an opposite dynamical pattern in the two heterografts (Opposite_heterograft), and genes specifically induced in the heterograft samples (Heterograft), the self-grafts (Homograft), the scion (Scion), or the stock (Stock). (C) Transcriptional regulations of three major TFs extracted from the heterograft GRNs that regulate more than 25 downstream targets in the pepper:tomato and tomato:pepper networks, including the graft-related ANAC071.

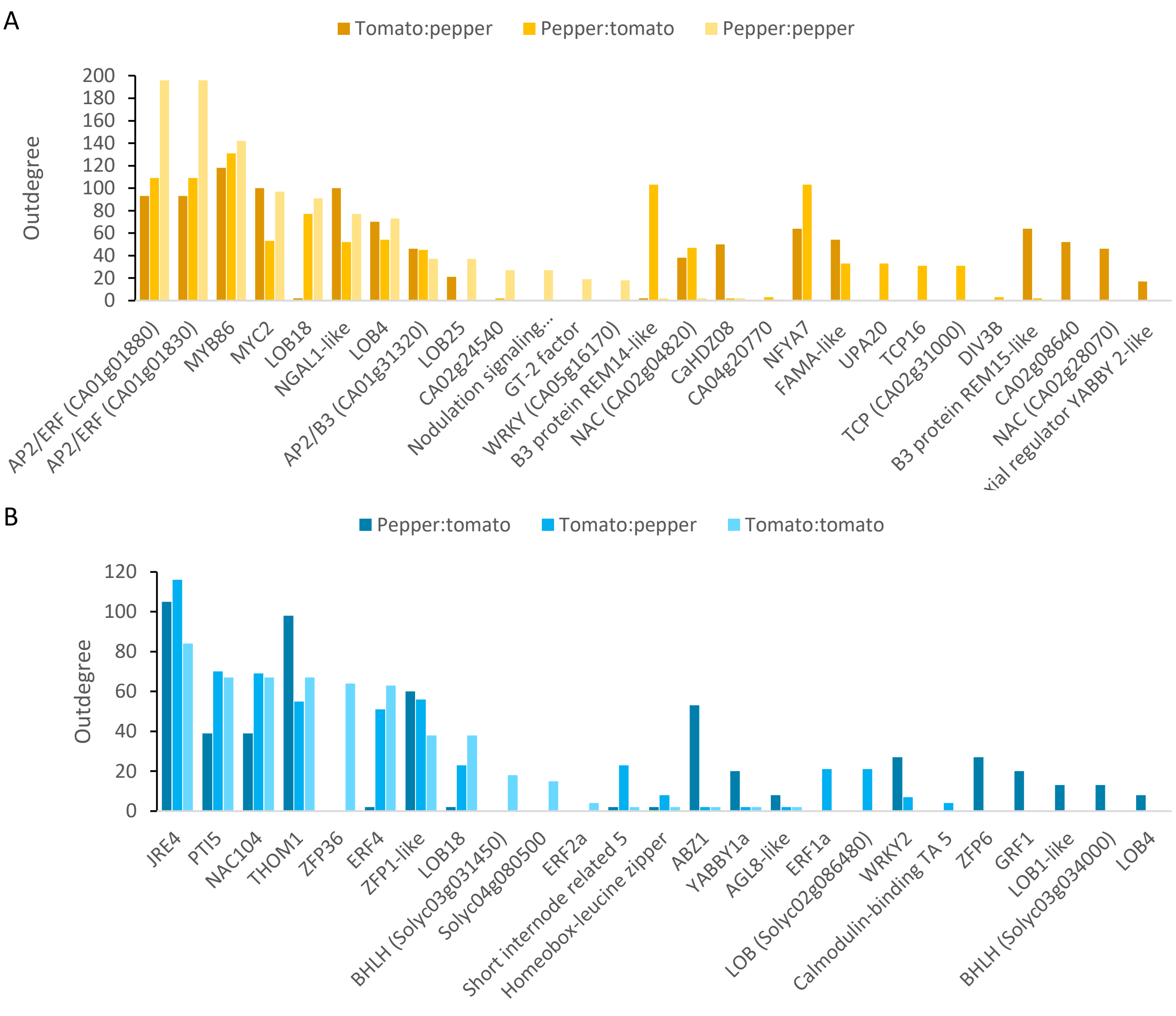

Supplemental Figure S11 Variation in outdegree in the self grafts and heterografts. (A-B) The outdegree is plotted of TFs with an outdegree > 2 for the pepper (A) and tomato genes (B).

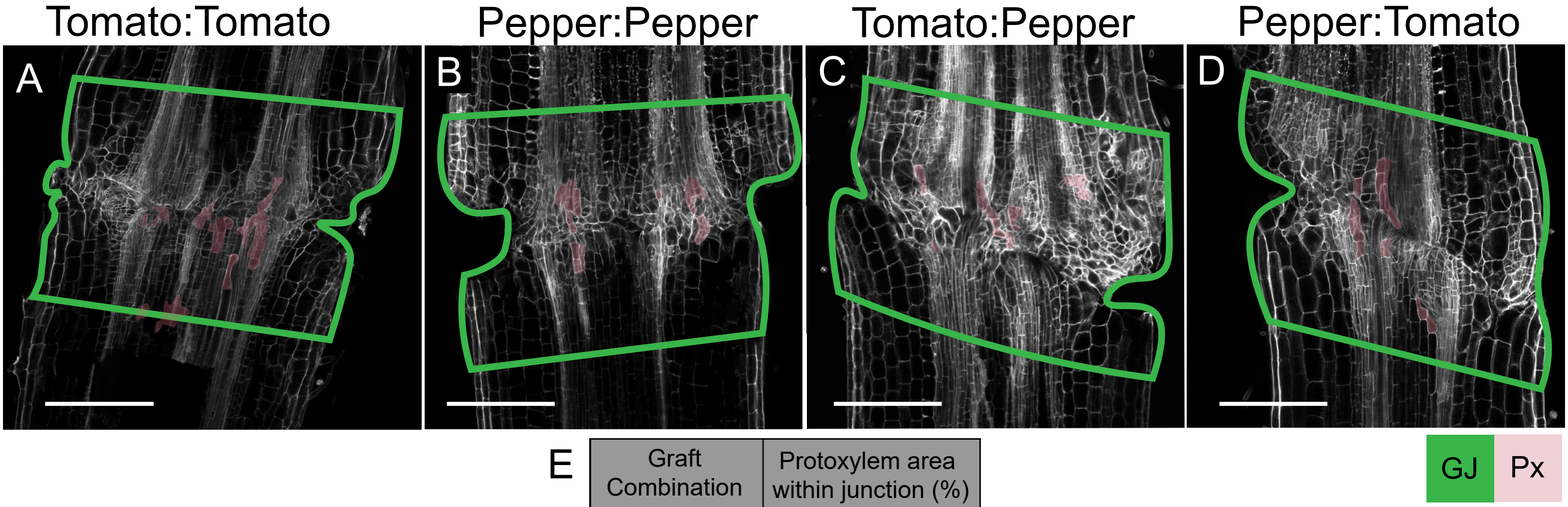

E

Graft Combination	Protoxylem area within junction (%)
T:T	1.88
P:P	1.18
T:P	1.13
P:T	1.27

Supplemental Figure S12 Protoxylem cells make up a minority of the newly formed tissue in the graft junction (Supports Figure 5). (A-D) Representative 2D area within 500 μm above and below the graft junction (GJ) in self grafted tomato (A), pepper (B), tomato:pepper (C) , and pepper:tomato (D). (E-H) Representative 2D area consisting of newly formed protoxylem (Px) cells 500 um above and below the graft junction in self grafted tomato (A), pepper (B), tomato:pepper (C) , and pepper:tomato (D). (I) Area within the 2D graft junction and protoxylem cells. Green shading signifies the graft junction (GJ). Pink shading signifies protoxylem cells (Px). Scale bars = 500 μm.

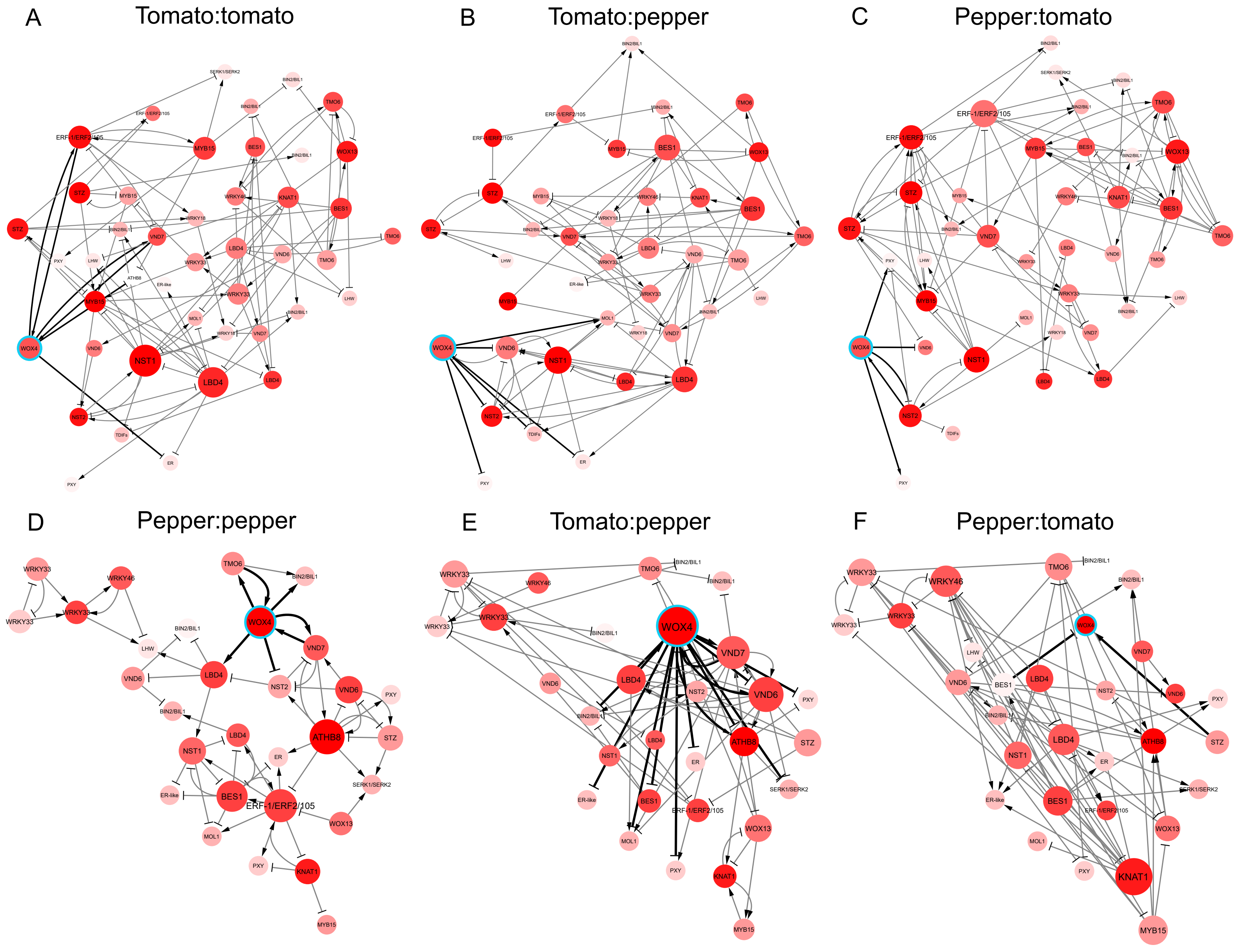

Supplemental Figure S13 WOX4 regulates xylem differentiation genes, *VND6/7* and *NST1/2*, in self-grafts and heterografts (Supports Figure 5). (A-C) The rewiring of 45 tomato genes in the tomato:tomato (A), tomato:pepper (B) and pepper:tomato (C) network is shown. (D-F) Similarly, the rewiring of 32 pepper genes in the pepper:pepper (D), tomato:pepper (E) and pepper:tomato (F) network is shown. Nodes are colored with different shades of red according to the magnitude of their variation in edge connections. The node and edges of WOX4 are highlighted with a blue border and bolded black arrows, respectively.

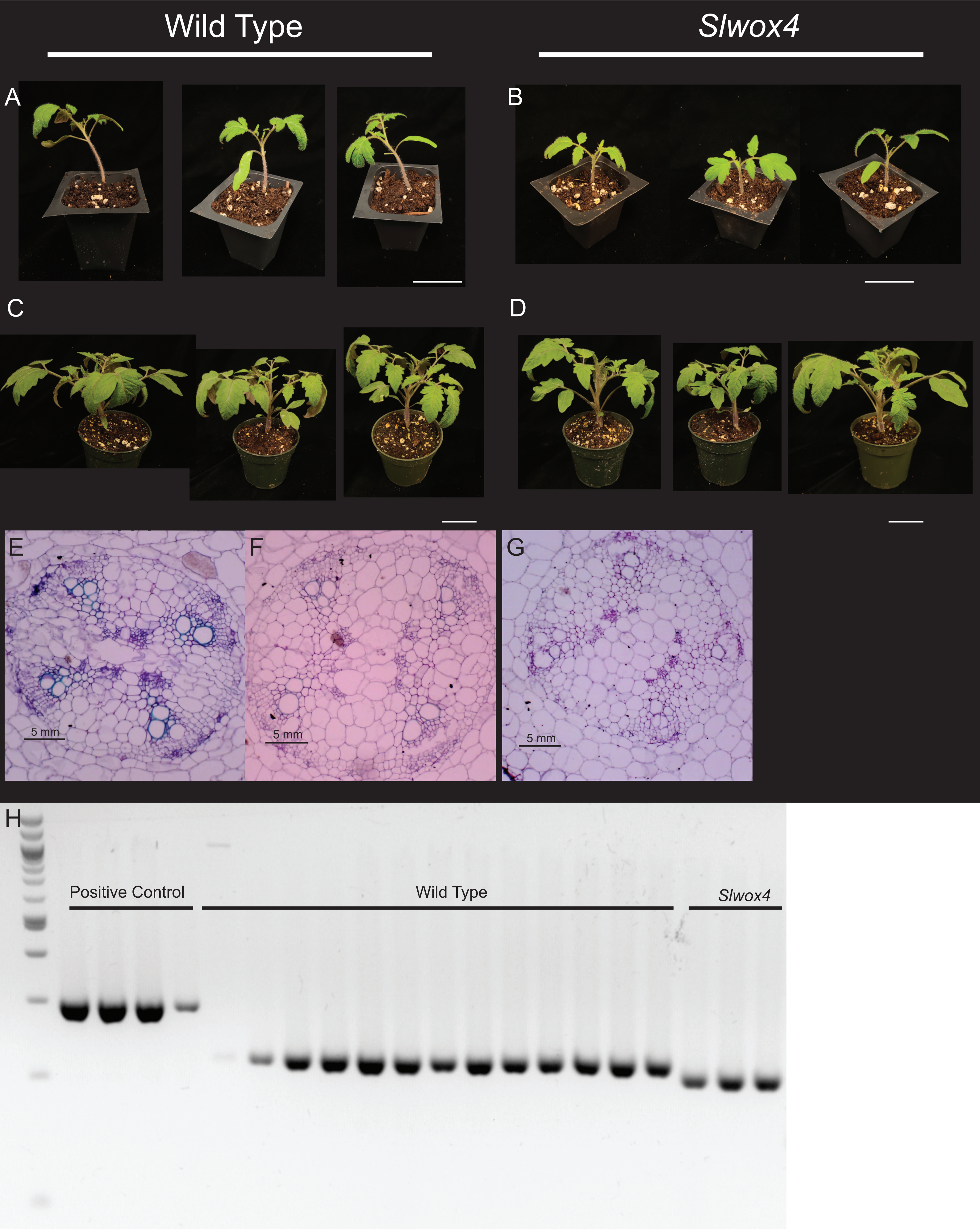

Supplemental Figure S14 *Slwox4* mutants exhibit minor alterations in vegetative phenotypes (Supports Figure 6). (A-D) Representative selection of wild type (WT) (A,C) and *Slwox4* (B,D) seedlings at the time of grafting (i.e. 3 weeks after imbibition) (A,B) and 5 weeks after imbibition (C,D). (E-G) Representative cross-sections sampled at time of grafting from the graft junction of WT (E,F) and *Slwox4* (G) plants. (H) PCR validation of homozygous 15 bp deletion in *Slwox4* mutants. Lane 1: 100 bp DNA ladder (NEB), Lanes 2-5: Positive controls (randomly selected WT and *Slwox4* DNA amplified with commercial positive control primer set from Thermo Scientific Phire Plant Direct PCR Kit, 297 bp), Lanes 6-18: WT DNA (223 bp), Lanes 19-21: *Slwox4* DNA (208 bp). scale bar = 6 cm (A,B), 9 cm (C,D), and 5 mm (E-G).

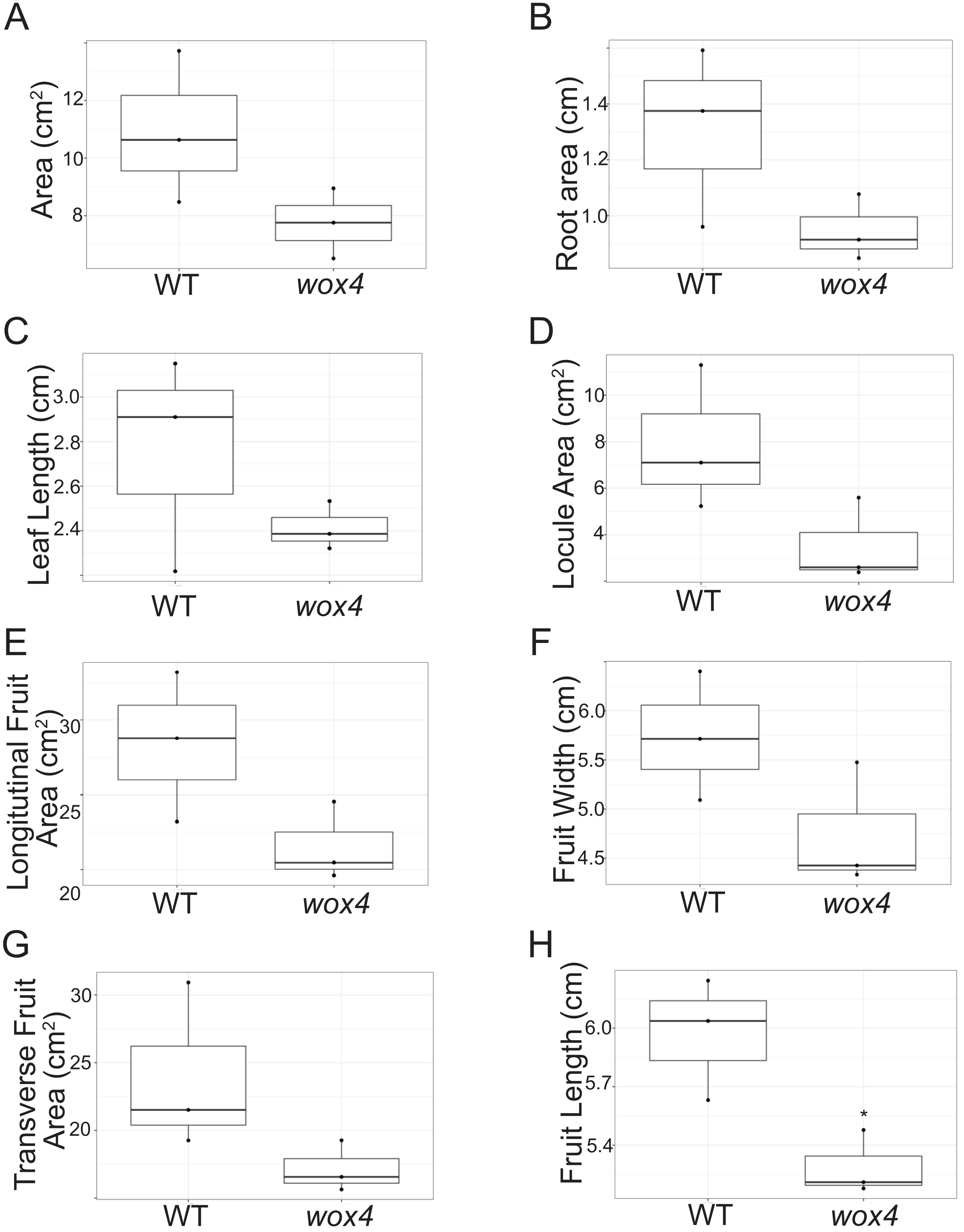

Supplemental Figure S15 Quantitative analysis of *Slwox4* vegetative and reproductive phenotypes (Supports Figure 6). (A-C) Quantification of the area (A), the root system area (B), and the length of the largest leaf (C) of 2-weeks-old wild type (WT) and *Slwox4* plants (3 weeks after imbibition). (D-H) Quantification of the area of the locule of mature fruit (transverse cut) (D), area of uncut mature fruit (side profile) (E), mature fruit width (F), area of mature fruit (transverse cut) (G), and mature fruit length (H) collected from WT and *Slwox4* plants. $p < 0.05$ = * (Student's t-test).

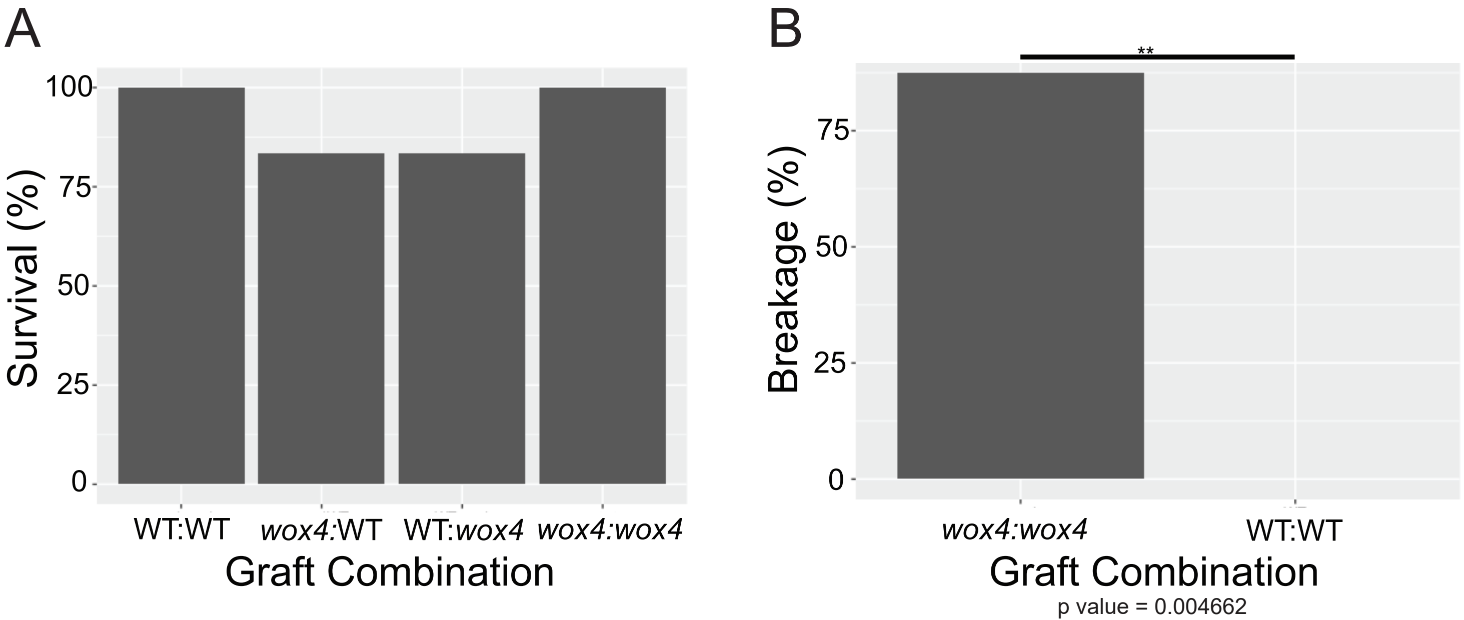

Supplemental Figure S16 ***Slwox4*** **mutant seedlings do not display decreased viability 30 DAG (Supports Figure 7).** (A) The percentage of surviving self-grafted WT and *Slwox4* plants 30 days after grafting (DAG). Fully wilted plants were considered dead. (B) The percentage of breakage along the graft site of self-grafted WT and *Slwox4* during bend tests. n=18, * = p-value < 0.05 (Fisher's Exact Test).

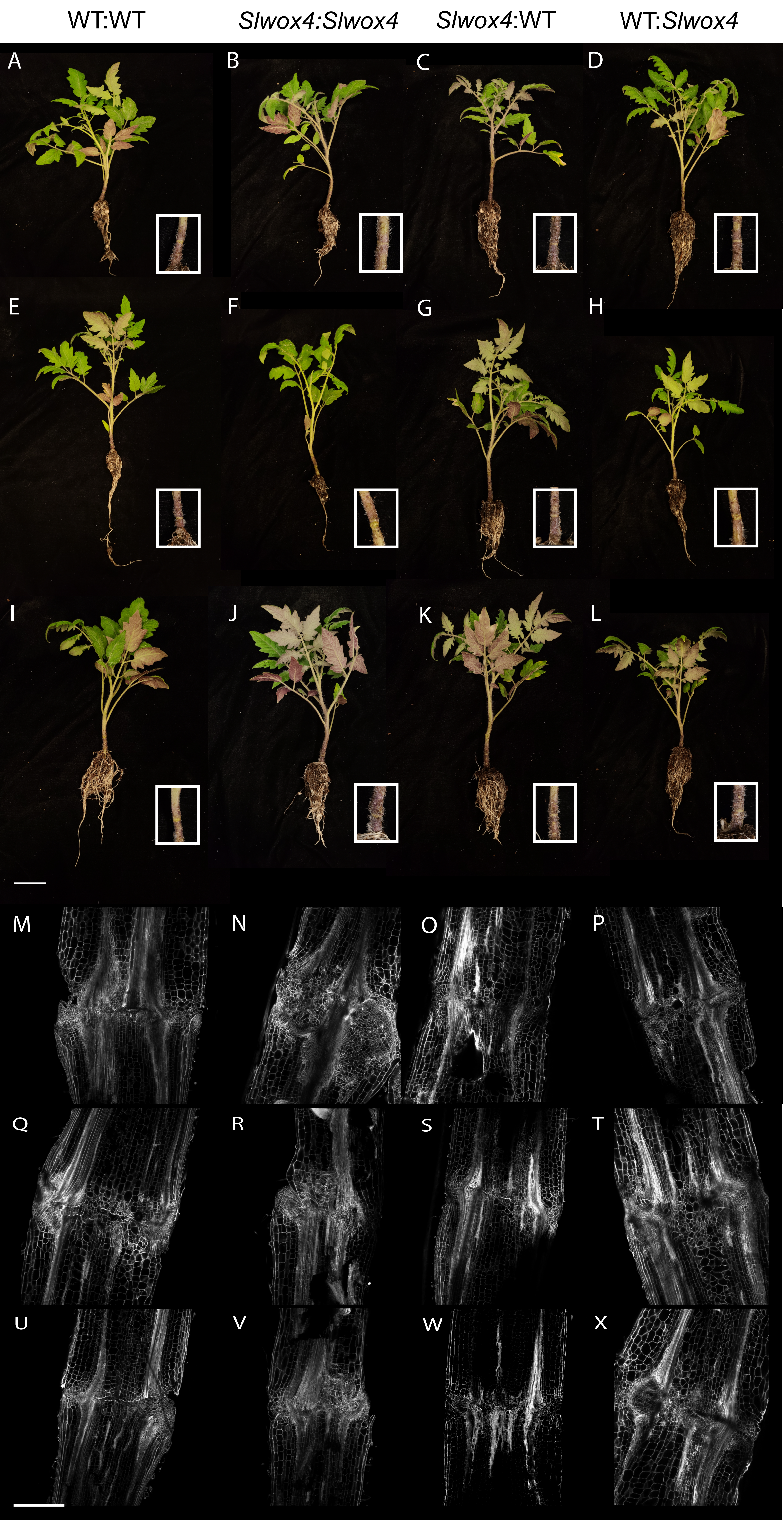

Supplemental Figure S17 Self-grafted *Slwox4* fail to form xylem bridges and thus exhibit graft incompatibility (Supports Figure 7). Representative images of self-grafted wild type (WT) (A,E,I,M,Q,U), self-grafted *Slwox4* (B,F,J,N,R,V), *Slwox4*:WT (C,G,K,O,S,W) WT:*Slwox4* (D,H,L,P,T,X) 30 days after grafting (DAG). Tissues in M-X were stained with propidium iodide and cleared in methyl salicylate. Scale bar = 5cm (A-L), and 800 µm (M-X).

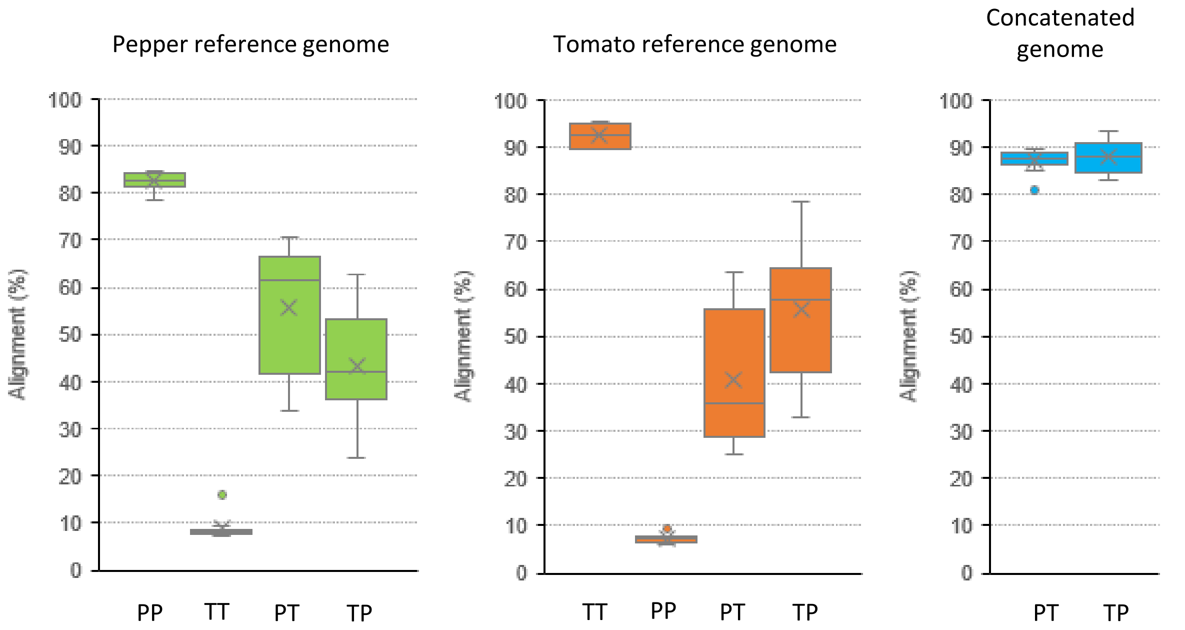

Supplemental Figure S18 Concatenated genome improves read alignment percentage for heterografted pepper and tomato (Supports Figure 3). Each sample, two self-grafts and two heterografts were aligned to the pepper (cvCM334) and tomato (*Solanum lycopersicum* cv Heinz) reference genome. The two heterografts were also aligned to a reference genome that concatenates the pepper and tomato reference genome (referred to as concatenated genome). PP = pepper:pepper self-graft, TT = tomato:tomato self-graft, PT = pepper:tomato heterograft, TP = tomato:pepper heterograft.

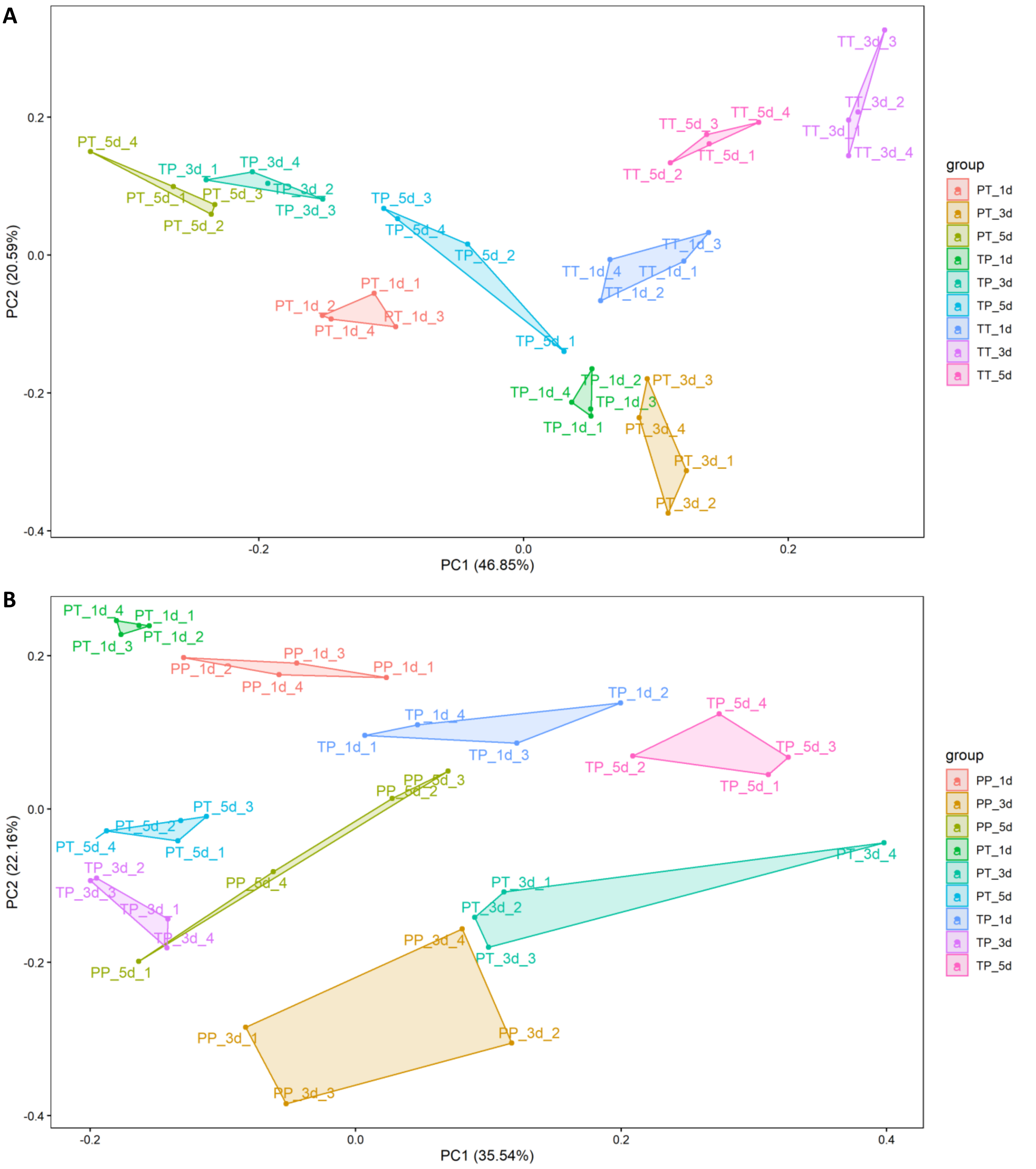

Supplemental Figure S19 Principal Component analysis (PCA) of the RNAseq samples (Supports Figure 3). (A,B) FPKM values of the tomato genes (A) and pepper genes (B) were used to perform PCA. In the PCA plot, each dot represents an RNAseq sample. The samples are plotted in two dimensions using their projections onto the first two principal components. PP = pepper:pepper self-graft, TT = tomato:tomato self-graft, PT = pepper:tomato heterograft, TP = tomato:pepper heterograft, PC = principal component.

Supplemental Table S1 Protoxylem area calculation 5 days after grafting (DAG). Column A lists the graft combination for each data point. Column B lists the total pixels found within the 1000 um piece of graft tissue. Column C lists the pixels within any hand-annotated protoxylem cells. Column D lists the percent of the total area that is protoxylem cells. Column E lists the average for each graft combination.

Graft Combination	Total pixel in 1000 um	protoxylem pixels	protoxylem/total (%)	Average
TT	1434823	60693	4.229999101	1.879984653
TT-2	674510	2564	0.380127796	
TT-3	543198	5594	1.029827061	
PP	1564210	47306	3.024274234	1.184967506
PP-2	543211	1296	0.238581325	
PP-3	467733	1366	0.292046958	
TP	1667049	42100	2.525420668	1.132791359
TP-2	440210	1547	0.351423184	
TP-3	496999	2592	0.521530224	
PT	1581895	51391	3.248698555	1.267959094
PT-2	521348	2876	0.551646885	
PT-3	527067	3130	0.593852394	
PT-4	488461	3310	0.677638542	

Supplemental Table S2 Phenotypic measurements of wild type and *Slwox4* vegetative and reproductive organs. The yellow table lists the raw data from three images per genotype for the following measurements (from left to right): 2-dimensional (2D) area of the 2-weeks-old plant, the length of the longest leaf, the 2D area of the root system from 2-weeks-old plants, the area of a transvere cut mature fruit, the 2D area the locule chambers, the 2D area of a fruit (side profile), the width of the fruit, and the length of the fruit. The blue table lists the output of the performed statistical analysis.

ID	2D Area (cm^2)	Length of longest leaf (cm)	Root Area (cm^2)	2D transverse fruit area (cm^2)	Locule area (cm^2)	Longitudinal fruit area (cm^2)
WT	8.476	3.15	0.96	19.26	5.229	23.2
WT	13.722	2.91	1.592	30.915	11.299	33.202
WT	10.632	2.218	1.375	21.513	7.101	28.786
wox4	6.514	2.386	0.848	16.568	2.38	19.594
wox4	8.949	2.533	1.077	15.639	2.596	20.46
wox4	7.757	2.321	0.914	19.266	5.594	24.54

ID	Fruit width (cm)	Fruit length (cm)
WT	5.091	5.631
WT	6.401	6.244
WT	5.714	6.037
wox4	4.332	5.178
wox4	4.425	5.478
wox4	5.475	5.21

Trait	Genotype	count	mean	SD	T.test	Percent Change
2D Area	WT	3	10.9	2.64	0.1581	-29%
	wox4	3	7.74	1.22		
Length of	WT	3	2.76	0.484	0.3407	-13%
	wox4	3	2.41	0.109		
Root Area	WT	3	1.31	0.321	0.1805	-28%
	wox4	3	0.946	0.118		
2D transv	WT	3	23.9	6.18	0.1927	-28%
	wox4	3	17.2	1.88		
Locule are	WT	3	7.88	3.11	0.1208	-55%
	wox4	3	3.52	1.8		
Longitudir	WT	3	28.4	5.01	0.1258	-24%
	wox4	3	21.5	2.64		
Fruit widtl	WT	3	5.74	0.655	0.133	-17%
	wox4	3	4.74	0.635		
fruit lengtl	WT	3	5.97	0.312	0.04331	-11%
	wox4	3	5.29	0.165		
